# Loss of myeloid Tsc2 predisposes to angiotensin II-induced aortic aneurysm formation in mice

**DOI:** 10.1038/s41419-022-05423-2

**Published:** 2022-11-18

**Authors:** Xue Liu, Yan Liu, Rui-xue Yang, Xiang-jiu Ding, Er-shun Liang

**Affiliations:** 1 The Key Laboratory of Cardiovascular Remodeling and Function Research, Chinese Ministry of Education, Chinese National Health Commission and Chinese Academy of Medical Sciences, The State and Shandong Province Joint Key Laboratory of Translational Cardiovascular Medicine, Department of Cardiology, Qilu Hospital of Shandong University, Jinan, China; 2 Department of Vascular Surgery, General Surgery, Qilu Hospital of Shandong University, Jinan, China

**Keywords:** Cardiovascular diseases, Experimental models of disease

## Abstract

**Rationale:**

Genetic studies have proved the involvement of Tuberous sclerosis complex subunit 2 (Tsc2) in aortic aneurysm. However, the exact role of macrophage Tsc2 in the vascular system remains unclear. Here, we examined the potential function of macrophage Tsc2 in the development of aortic remodeling and aortic aneurysms.

**Methods and results:**

Conditional gene knockout strategy combined with histology and whole-transcriptomic analysis showed that Tsc2 deficiency in macrophages aggravated the progression of aortic aneurysms along with an upregulation of proinflammatory cytokines and matrix metallopeptidase-9 in the angiotensin II-induced mouse model. G protein-coupled receptor 68 (Gpr68), a proton-sensing receptor for detecting the extracellular acidic pH, was identified as the most up-regulated gene in Tsc2 deficient macrophages compared with control macrophages. Additionally, Tsc2 deficient macrophages displayed higher glycolysis and glycolytic inhibitor 2-deoxy-D-glucose treatment partially attenuated the level of Gpr68. We further demonstrated an Tsc2-Gpr68-CREB network in macrophages that regulates the inflammatory response, proteolytic degradation and vascular homeostasis. Gpr68 inhibition largely abrogated the progression of aortic aneurysms caused by Tsc2 deficiency in macrophages.

**Conclusions:**

The findings reveal that Tsc2 deficiency in macrophages contributes to aortic aneurysm formation, at least in part, by upregulating Gpr68 expression, which subsequently drives proinflammatory processes and matrix metallopeptidase activation. The data also provide a novel therapeutic strategy to limit the progression of the aneurysm resulting from Tsc2 mutations.

## Introduction

Aortic aneurysm (AA) is a life-threatening aortic disease, characterized by a progressive dilatation of the aorta. A majority of AAs are located at two distinct aneurysm-prone regions, the abdominal aorta and thoracic aorta involving the ascending aorta. Small-sized AAs are mostly asymptomatic, whereas large-sized AAs often lead to aortic rupture and sudden death when the diameters are more than 6.0 cm in thoracic aortic aneurysm (TAA) and more than 5.0 cm in abdominal aortic aneurysm (AAA) [1, 2]. Although great progress has been achieved in surgical techniques including endovascular aneurysm repair, there is still no effective pharmacological treatments for patients with small AAs or contradictions to surgery [3]. Thus, it is important to expand our knowledge of the mechanisms involved in AAs development and progression.

Although TAA and AAA have different aetiologies and pathogeneses, accumulating experimental evidence propose the strong role of a chronic immune/inflammatory process in both TAA and AAA evocation and progression [3, 4]. Likewise to AAA, histological studies demonstrate the presence of large amounts of inflammatory cell infiltrates, particularly macrophages, in the media and adventitia of thoracic aortic wall [4]. Infiltrating macrophages contribute to the degradation of extracellular matrix and death or dysfunction of vascular smooth muscle cells (SMCs) by releasing a range of proteolytic enzymes, including matrix metalloproteinases (MMPs), oxidation-derived free radicals and proinflammatory cytokines (TNF-α, IL-1β, and IL-6), which induce arterial wall destruction and subsequent aorta dilatation [3, 4].

Tuberous sclerosis complex subunit 1 (Tsc1) and 2 (Tsc2), which encode the proteins hamartin and tuberin, respectively, form a heterodimeric complex. Tsc1 enhances Tsc2 function by stabilizing Tsc2. Tsc complex participates in a wide range of cellular functions and acts as a tumor growth suppressor [5]. Studies have shown that Tsc2 is a catalytic GTPase-activating protein (GAP) and directly inhibits Rheb small GTPase upstream of mTOR [6, 7]. Inactivating mutations in Tsc1 or Tsc2 give rise to the tuberous sclerosis. Patients with tuberous sclerosis have a higher incidence of AAs compared with the general population [8]. However, the underlying molecular mechanisms are poorly understood. Recent studies have reported the role of smooth muscle Tsc1/Tsc2 in the pathogenesis of AAs [9, 10]. Tsc1 deletion in smooth muscle cells results in a degradative smooth muscle cell phenotype and progressive TAA [9]. In addition, descending aortic aneurysm is associated with subintimal smooth muscle cell proliferation in a child with a Tsc2 mutation [10]. Whether the deficiency of macrophage Tsc2 is directly involved in aneurysm formation is still a mystery. Emerging evidence indicates that macrophage Tsc2 orchestrates inflammatory response, implying that macrophage Tsc2 may be closely related to vascular homeostasis [11, 12]. Thus, we aimed to evaluate the role of macrophage Tsc2 in vascular inflammation and aneurysm pathogenesis.

In this study, we demonstrate that myeloid Tsc2 depletion increased susceptibility to AAs formation and elastin degradation in the angiotensin II-induced mouse model. An evaluation for the underlying mechanism of exacerbating vascular inflammation and extracellular matrix remodeling in myeloid-specific Tsc2 deficient mice leads to the identification of the macrophage G protein-coupled receptor 68 (Gpr68) as a key molecule involved in AAs formation.

## Results

### Myeloid-specific deletion of Tsc2 exacerbates AngII (angiotensin II) infusion–induced aneurysm development

Animals with myeloid-specific disruption of Tsc2 were sacrificed and their aortas compared with controls. Conditional myeloid deletion of Tsc2 in an ApoE^–/–^ background induced swollen paws and tails (Fig. S1A and B), which was accordance with a previous report [11]. However, Tsc2^MKO^ApoE^–/–^ mice are viable and fertile with uncompromised vasculature development. Systemic infusion of AngII via osmotic mini-pumps into ApoE^–/–^ mice for 4 weeks has been repeatedly demonstrated to cause aneurysmal pathology in both thoracic and abdominal aortas [13]. Subsequently, to investigate whether macrophage Tsc2 is crucial to maintain vasculature homeostasis, we crossed myeloid-specifc Tsc2^MKO^ with hyperlipidemic ApoE^–/–^ mice and established AngII-induced murine aortic aneurysm model. The absence of Tsc2 expression on myeloid cells in Tsc2^MKO^ApoE^–/–^ mice was confirmed by RT-PCR and western blot analysis in peritoneal macrophages (Fig. S1C and D).

**Fig. 1:**
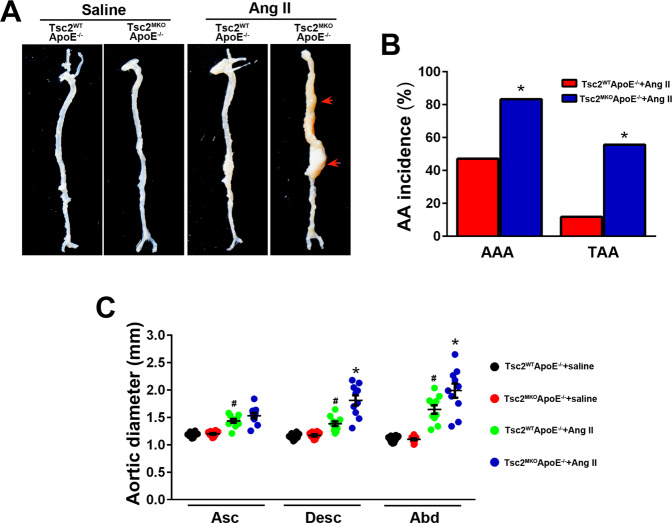
Myeloid-specific deletion of Tsc2 exacerbates AngII infusion-induced aortic aneurysms in vivo. Tsc2^MKO^ApoE^−/−^ and Tsc2^WT^ApoE^−/−^ mice were infused with saline or AngII for 28 d. **A** Representative images of aortic specimens from saline or AngII-infused Tsc2^WT^ApoE^−/−^ and Tsc2^MKO^ApoE^–/–^ mice. **B** Incidence of AAA and TAA in AngII-infused Tsc2^WT^ApoE^–/–^ and Tsc2^MKO^ApoE^–/–^ mice (Tsc2^WT^ApoE^–/–^ + saline, *n* = 10; Tsc2^MKO^ApoE^–/–^ + saline, *n* = 10; Tsc2^WT^ApoE^−/−^ + AngII, *n* = 17; Tsc2^MKO^ApoE^–/–^ + AngII, *n* = 18). **C** Comparison of maximal diameters in the ascending thoracic (Asc), descending thoracic (Desc), and suprarenal abdominal (Abd) aortic segments measured with vernier caliper (*n* = 10). *P < 0.05, vs. Tsc2^WT^ApoE^–/–^ mice infused with AngII. ^#^*P* < 0.05, vs^.^ Tsc2^WT^ApoE^–/–^ mice infused with saline.

After 4 weeks of AngII (1000 ng/kg/min) infusion, systolic blood pressure increased similarly in conscious Tsc2^MKO^ApoE^–/–^ and Tsc2^WT^ApoE^–/–^ mice, but no significant difference in body weight between the two groups was observed (Table S1). Morphologically, the aortas of saline-infused Tsc2^MKO^ApoE^–/–^ mice did not differ from those of saline-infused controls (Fig. 1A). No spontaneous aortic dilations and aneurysms were observed in the saline-infused mice (Fig. 1A). Although AngII treatment resulted in vascular expansion in both genotypes, Tsc2^MKO^ApoE^–/–^ mice exhibited significant increases in the maximal descending thoracic and abdominal aortic diameter compared to Tsc2^WT^ApoE^–/–^ mice (Fig. 1A–C). As well, both the incidence of AAA and TAA were markedly increased in Tsc2^MKO^ApoE^−/−^ mice (83.3% and 55.6%, respectively) than in Tsc2^WT^ApoE^–/–^ mice (47.1% and 11.8%, Fig. 1B, Table S2).

### Myeloid-specific deletion of Tsc2 promotes AngII infusion–induced aortic structural and composition changes

**Fig. 2:**
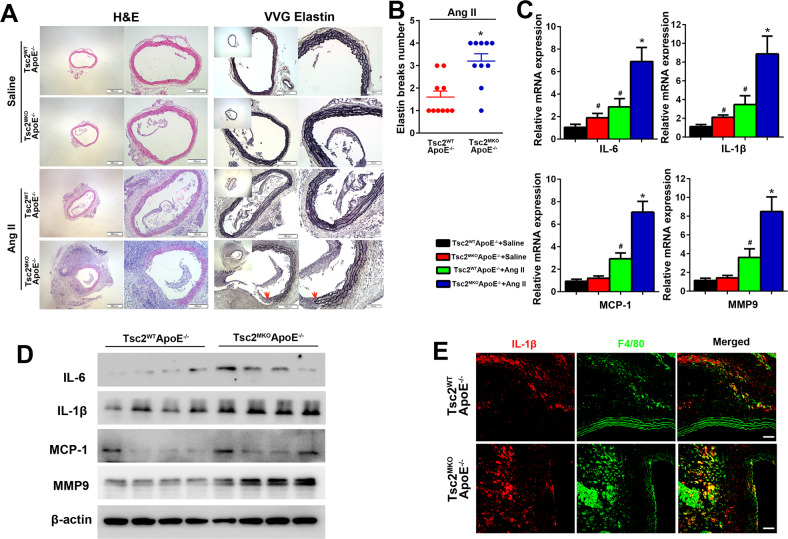
Myeloid-specific deletion of Tsc2 exacerbates aortic elastic fiber degradation and vascular inflammation in AngII infused mice. **A** Representative hematoxylin and eosin (H&E) and Verhoeff-van Gieson (VVG) staining of the abdominal aortic sections in Tsc2^WT^ApoE^–/–^ and Tsc2^MKO^ApoE^–/–^ mice with or without AngII infusion. Red arrow indicated elastin break. **B** Quantifcation of elastin breaks per section in aortic cross sections harvested from AngII infused mice (*n* = 10). **C** Quantification of IL-6, IL1-β, MCP-1, and Mmp9 mRNA expression in abdominal aorta harvested from Tsc2^WT^ApoE^–/–^ and Tsc2^MKO^ApoE^–/–^ mice with or without AngII infusion for 28 d (*n* = 5). **D** Western blot analysis of IL-6, IL1-β, MCP-1, and Mmp9 expression in abdominal aorta harvested from AngII-infused Tsc2^WT^ApoE^–/–^ and Tsc2^MKO^ApoE^–/–^ mice (*n* = 4). **E** Representative images of IL1-β expression by F4/80^+^ macrophages in abdominal aortic tissues from AngII-infused Tsc2^MKO^ApoE^–/–^ and Tsc2^WT^ApoE^–/–^ mice (*n* = 5). *P < 0.05, vs. Tsc2^WT^ApoE^–/–^ mice infused with AngII. ^#^*P* < 0.05, vs^.^ Tsc2^WT^ApoE^–/–^ mice infused with saline.

Elastin fragmentation is considered crucial for aortic wall dilation and can be detected by Verhoeff-Van Giessen (VVG) staining. Histological analysis of the suprarenal aorta with VVG staining showed that elastin fibers were disrupted in AngII-infused arteries (Fig. 2A, B). No obvious elastin breakage was identified in aortic sections from saline-infused Tsc2^MKO^ApoE^−/–^ and Tsc2^WT^ApoE^–/–^ mice. Notably, disruption and degradation of medial elastic lamina were more severe in AngII-infused Tsc2^MKO^ApoE^–/–^ mice compared with Tsc2^WT^ApoE^–/–^ mice (Fig. 2A, B).

We then checked the expression of several proinflammatory cytokines and matrix metalloproteinase-9 (Mmp9), which have been repeatedly demonstrated to contribute to the pathogenesis of AAs in the aortas [14]. Tsc2 deletion in myeloid cells significantly increased the expression of IL-6 and IL1-β but not MCP-1 and Mmp9 expression in the saline-infused aortas compared with control mice (Fig. 2C). In addition, AngII augmented the expression of proinflammatory cytokines IL-6, IL1-β and MCP-1 in the presence as well as in absence of Tsc2 (Fig. 2C and D). Consistently, the aortas from Tsc2^MKO^ApoE^−/−^ mice infused with AngII displayed a marked upregulation of Mmp9 expression (Fig. 2C and D). Furthermore, double immunofluorescence analysis detected increased F4/80^+^ macrophages infiltrating into aortas expressed IL1-β in the aortas from AngII-infused Tsc2^MKO^ApoE^−/−^ mice (Fig. 2E).

**Fig. 3:**
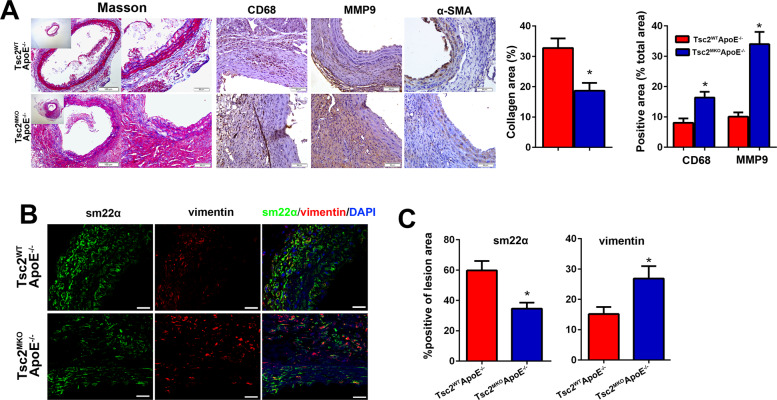
Myeloid-specific deletion of Tsc2 promotes AngII infusion–induced aortic structural and composition changes. **A** Masson and immunohistochemical staining of the abdominal aortic sections for CD68, α-SMA, and Mmp9 in Tsc2^WT^ApoE^–/–^ and Tsc2^MKO^ApoE^–/–^ mice receiving AngII infusion for 28 d (*n* = 5). **B** Immunofluorescence staining of sm22α (green) and vimentin (red) in abdominal aortic sections (*n* = 5). **C** Quantification of sm22α and vimentin coverage (*n* = 5). Scale bar = 50 µm. *P < 0.05, vs. Tsc2^WT^ApoE^–/–^ mice infused with AngII.

Moreover, Tsc2 deletion in myeloid cells decreased the relative contents of collagen and smooth muscle cells but augmented macrophage infiltration in the aortic wall, as determined by Masson’s trichrome staining and immunostaining (Fig. 3A). As proinflammatory macrophages could enhance the phenotypic changes of smooth muscle cells [15] and thus aggravate aortic aneurysm development [16], we performed co-immunostaining of mouse abdominal aortic tissues. As revealed by immunofluorescence staining (Fig. 3B and C), Tsc2 deletion in myeloid cells significantly decreased the expression of contractile SMC phenotype marker sm22α but increased the synthetic phenotype marker vimentin in aneurysm tissues. These observations would indicate that the lack of myeloid Tsc2 could aggravate AngII-induced aortic inflammatory responses and structural remodeling.

### Identification of Gpr68 up-regulation in Tsc2 deficient macrophages following AngII infusion

**Fig. 4:**
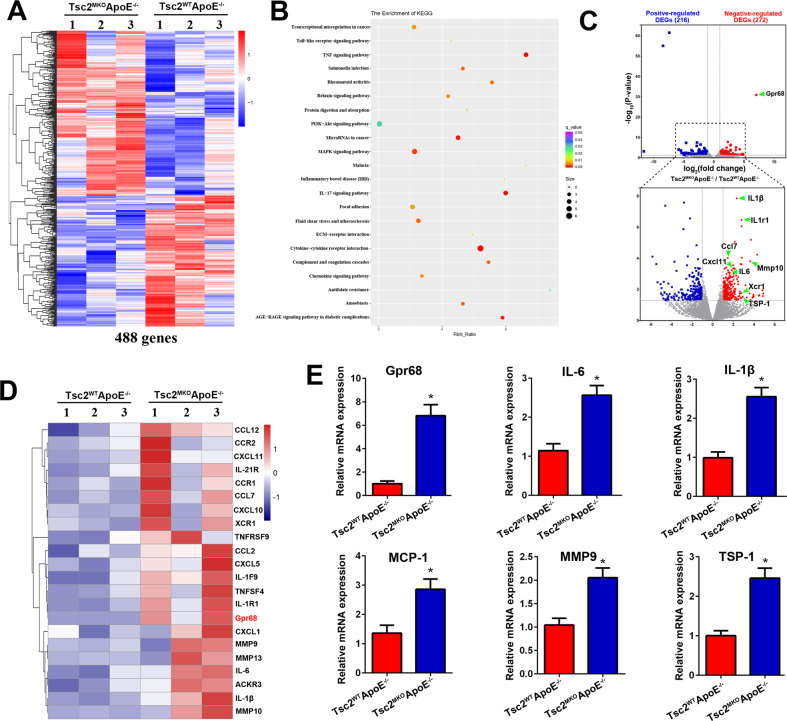
Identification of target genes regulated by Tsc2 with genome-wide RNA-seq analysis. **A** Heat map displayed the top genes with altered expression (|log2FC | >1) in the peritoneal macrophages from Tsc2^MKO^ApoE^–/–^ mice versus Tsc2^WT^ApoE^–/–^ infused with AngII for 7 d. **B** KEGG pathway enrichment analysis was conducted. **C** Valconoplot shows genes whose expression was significantly altered upon Tsc2 deletion. Blue plots represented significant positive-regulated genes and the red plots for negative-regulated genes. **D** Heat map of the selected genes from (**A**). **E** Quantitative real-time PCR were performed to evaluate mRNA expression of the indicated genes (*n* = 5). *P < 0.05, vs. macrophages from Tsc2^WT^ApoE^–/–^ mice infused with AngII.

To clarify the mechanism whereby aneurysm development was exaggerated in AngII-infused Tsc2^MKO^ApoE^–/–^ mice, we first identified the genes that are regulated by Tsc2 in macrophages following AngII infusion. We performed RNA-Seq analysis on the peritoneal macrophages from Tsc2^MKO^ApoE^–/–^ mice versus Tsc2^WT^ApoE^–/–^ infused with AngII for **7** days. As a result, 488 genes were found to be differentially regulated by Tsc2 inactivation (twofold change in expression level; *P* < 0.05) (Fig. 4A, Table S3). Kyoto Encyclopedia of Genes and Genomes (KEGG) enrichment analysis was conducted on the genes related to Tsc2 expression and revealed that the genes were mainly enriched in several inflammation-related signaling pathways, such as cytokine-cytokine receptor interaction, the TNF signaling pathway and IL-17 signaling (Fig. 4B). Multiple pathways relevant to inflammation are activated in macrophages from Tsc2^MKO^ApoE^–/–^ mice.

The volcano plot of differentially expressed genes (DEGs) is shown in Fig. 4C. We identified 216 positive-regulated DEGs, which were significantly downregulated in Tsc2 deficient macrophages. And 272 genes were negative-regulated DEGs, which were significantly upregulated in Tsc2 deficient macrophages (Fig. 4C). Among them, we found 19 upregulated genes were associated with immune and inflammatory responses, including IL-6, IL-1β, MCP-1/Ccl2 (C-C motif chemokine ligand 2), Ccl7, Ccl12. Besides, MMP family members (Mmp9, Mmp10, Mmp13), Fos family members (Fosl1, Fos) and TSP-1 (thrombospondin-1) also showed significantly induction with Tsc2 deletion (Fig. 4C, Table S3), consistent with enhanced inflammatory responses in Tsc2 deficient macrophages and the consequent impact on AAs formation.

Notably, Gpr68, a proton-sensing receptor for detecting the extracellular acidic pH which has increased potential for producing inflammatory cytokines [17], was identified as the most up-regulated gene in Tsc2 deficient macrophages compared with control macrophages (Fig. 4C and D). A database inquiry indicated that Gpr68 was expressed ubiquitously, with relatively high expression in lipopolysaccharide-induced pro-inflammatory macrophages (Fig. S2A). These changes in the mRNA expressions were further confirmed by RT-PCR analysis (Fig. 4E). To investigate the potential mechanisms underlying Tsc2-mediated macrophage inflammation in vivo during aortic aneurysm formation, we isolated aortic macrophages from digested whole aorta using a magnetic-activated cell sorting system on day 28 from mice infused with AngII as previously described [18, 19]. Consistent with the studies in peritoneal macrophages, Gpr68 and proinflammatory cytokines (IL-6, IL-1β, MCP-1) expression were increased in aortic CD11b^+^ myeloid cells from AngII-infused Tsc2^MKO^ApoE^–/–^ mice in comparison to controls (Fig. S2B). Taken together, these findings from RNA-seq substantiate a crucial role for Tsc2 in regulating inflammatory phenotype of macrophages.

### Myeloid-specific deletion of Tsc2 induces Gpr68 expression in AngII-infused aortas

**Fig. 5:**
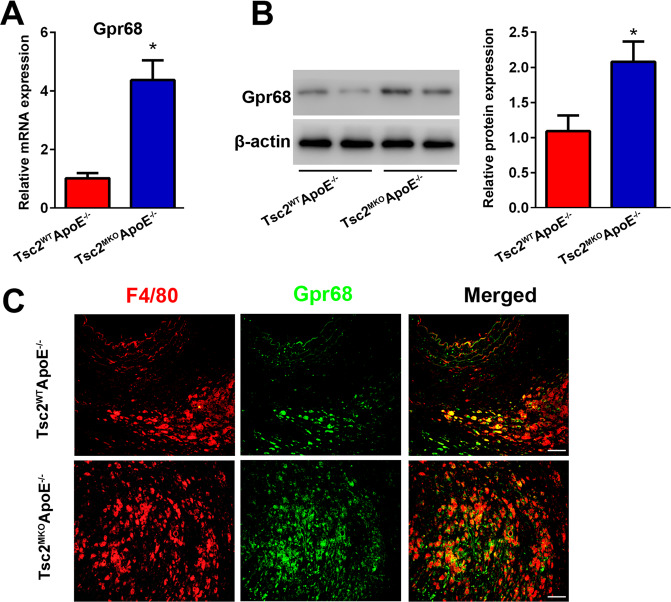
Myeloid-specific deletion of Tsc2 induces Gpr68 expression in AngII-infused aortas. **A**, **B** Quantitative real-time PCR (*n* = 5) and western blot (*n* = 4) were performed to determine the expression of Gpr68 in abdominal aortas from AngII-infused Tsc2^MKO^ApoE^–/–^ and Tsc2^WT^ApoE^–/–^ mice on day 28. **C** Immunofluorescence staining of F4/80 (red) and Gpr68 (green) in abdominal aortic tissues from AngII-infused Tsc2^MKO^ApoE^–/–^ and Tsc2^WT^ApoE^–/–^ mice on day 28 (*n* = 5). Scale bar = 50 µm. *P < 0.05, vs. Tsc2^WT^ApoE^–/–^ mice infused with AngII.

To further unravel the potential contribution of Tsc2 deletion in the aortic response to AngII, Gpr68 expression was detected in AngII-infused aortas. Immunoblotting and RT-PCR analysis confirmed the upregulation of Gpr68 in aortas from AngII-infused Tsc2^MKO^ApoE^–/–^ mice compared with control mice (Fig. 5A and B). Furthermore, increased F4/80^+^ macrophages infiltrating into aortas expressed Gpr68 in Tsc2^MKO^ApoE^–/–^ mice, as revealed by immunofluorescence staining (Fig. 5C). These results suggest that myeloid-specific deletion of Tsc2 induces high Gpr68-expressing macrophages in AngII-infused aortas.

We next examined how Tsc2 regulates the expression of Gpr68 in macrophages. As Gpr68 responds to extracellular acidosis, we measured the cellular oxygen consumption rate (OCR) and extracellular acidification rate (ECAR) in the peritoneal macrophages from AngII-infused mice. Consistent with the increased expression of Gpr68, Tsc2 deficient macrophages displayed higher glycolysis and mitochondrial respiration, as evidenced by increased ECAR and OCR (Fig. S3A and B). Compared with wild-type macrophages, Tsc2 deficient macrophages showed significantly increased migration (Fig. S3C). Of note, 2-deoxy-D-glucose (2-DG, 2 mg/mL) treatment partially attenuated the expression of Gpr68, indicating that this increased Gpr68 expression seemed to be mediated partially by the enhanced glycolytic activity induced by Tsc2 deficiency (Fig. S3D).

### Downregulation of Gpr68 alleviates the expression of inflammatory cytokines and Mmp9 in Tsc2 deficient macrophages

**Fig. 6:**
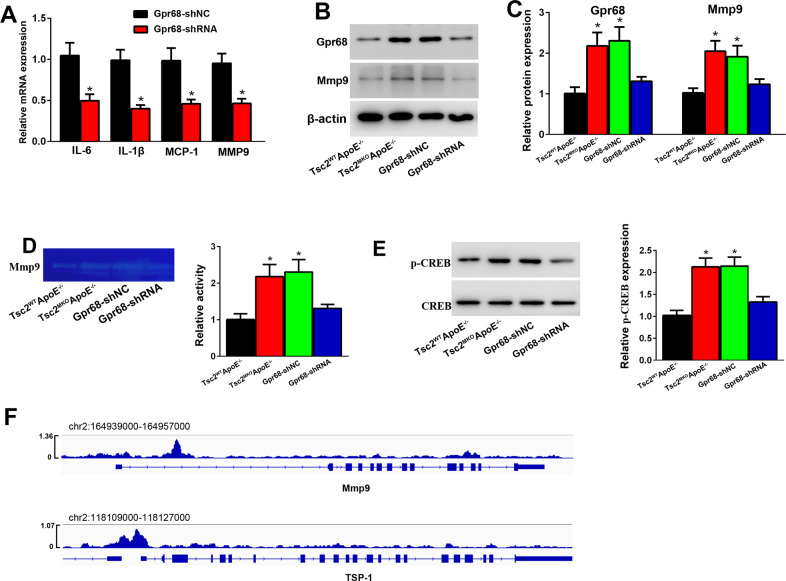
Gpr68 inhibition alleviates the expression of inflammatory cytokines and Mmp9 in Tsc2 deficient macrophages. Macrophages from AngII-infused Tsc2^MKO^ApoE^–/–^ mice were treated with shRNA-Gpr68 or negative control for 24 h. **A** Quantitative real-time PCR analysis of IL-6, IL1-β, MCP-1, and Mmp9 mRNA expression (*n* = 5). **B**, **C** Western blot analysis of Mmp9 and Gpr68 protein expression (*n* = 4). **D** Gelatin zymography analysis of Mmp9 activity (*n* = 4). **E** Western blot analysis of phosphorylated CREB expression (*n* = 4). *P < 0.05, vs. macrophages treated with negative control or macrophages from AngII-infused Tsc2^WT^ApoE^–/–^ mice. **F** Chromatin immunoprecipitation-seq assay of the occupancy of Mmp9 and TSP-1 gene promoters by CREB (data from GSM2663863 dataset).

To further detect the role of elevated expression of Gpr68, macrophages from AngII-infused Tsc2^MKO^ApoE^−/−^ mice were transfected with shRNA-Gpr68 or negative control shRNA (shRNA-NC) for 24 h. As shown in Fig. 6A, Gpr68 inhibition decreased the expression of proinflammatory cytokines IL-6, IL-1β and MCP-1. In addition, Gpr68 inhibition significantly suppressed Mmp9 expression and activity, as determined by western blot and zymography (Fig. 6B-D).

Gpr68 reportedly can couple to heterotrimeric G proteins and mediates the cAMP-PKA (protein kinase A)-CREB (cAMP response element-binding protein) pathway [20]. CREB functions as a transcription factor that can specifically bind to the cAMP response element (CRE) present in the promoter regions of downstream target genes [21]. Positive immunostaining of phosphorylated CREB has been observed in macrophages of AAs tissues [22], and the modulation of CREB on Mmp9 expression in macrophages has been reported [23], as well. Additionally, recent study has shown that CREB promotes transactivation of Fos gene family (Fosb, Fosl1, c-Fos) to enhance macrophage-mediated inflammation by directly binding to the promoters of these genes [24]. CREB also modulates the expression of TSP-1 [25], a member of the matricellular thrombospondin protein family which plays a key role in the regulation of macrophage inflammatory response and gelatinase activity during the pathogenesis of AAs [26]. We then further investigated the role of Gpr68 in the regulation of CREB activity. In macrophages from AngII-infused Tsc2^MKO^ApoE^−/−^ mice, the level of phosphorylated CREB were increased than in macrophages from AngII-infused Tsc2^WT^ApoE^−/−^ mice (Fig. 6E). Gpr68 inhibition reduced the level of phosphorylated CREB (Fig. 6E). Morever, CREB bound to the promoter region of Mmp9 and TSP-1 in murine bone marrow-derived macrophages according to the ChIP assay (Fig. 6F, source data obtained from GEO data set, GSM2663863). These data suggest that Tsc2 may mediates gene transcriptional activation through Gpr68/CREB pathway.

### Downregulation of Gpr68 prevents aneurysm formation induced by AngII administration in Tsc2^MKO^ApoE^−/−^ mice

**Fig. 7:**
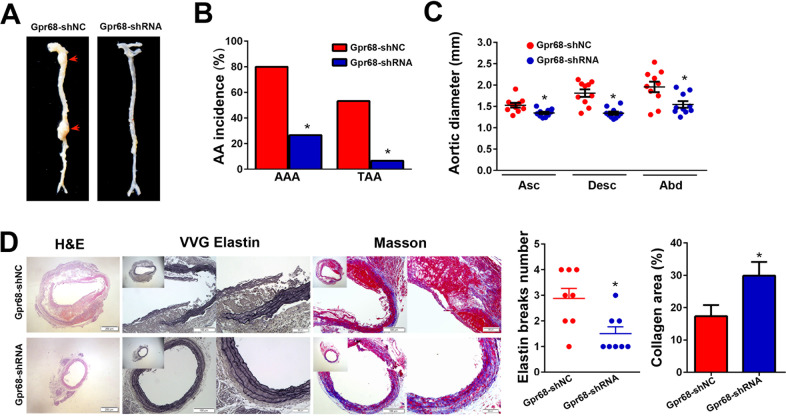
Gpr68 inhibition prevents aneurysm formation induced by AngII administration in Tsc2^MKO^ApoE^–/–^ mice. Tsc2^MKO^ApoE^–/–^ mice were treated with shRNA-Gpr68 or negative control before AngII pump implantation. At 28 days after the osmotic pumps implantation, mice were sacrificed, and their aortas were obtained. **A** Representative images of aortic specimens from shRNA-Gpr68 or negative control treated mice. **B** Incidence of AAA and TAA in shRNA-Gpr68 or negative control treated mice (*n* = 15). **C** Comparison of maximal diameters in the ascending thoracic (Asc), descending thoracic (Desc), and suprarenal abdominal (Abd) aortic segments measured with vernier caliper (*n* = 10). **D** Representative hematoxylin and eosin (H&E), Verhoeff-van Gieson (VVG), and Masson staining of the abdominal aortic sections. Quantifcation of elastin breaks per section in aortic cross sections (*n* = 8). Quantitative analysis of Masson staining signals (*n* = 5). *P < 0.05, vs. mice treated with negative control.

To investigate whether high Gpr68-expressing macrophages are involved in the exacerbation of AngII-induced AAs development, Tsc2^MKO^ApoE^−/−^ mice were randomized to receive shRNA-Gpr68 or shRNA-NC before AngII pump implantation. Gpr68 inhibition resulted in a marked reduction in AAs incidence and diameter (Fig. 7A-C, Table S2) without affecting blood pressure levels (Table S1). Histological analysis showed that aortas from shRNA-Gpr68 treated mice exhibited a moderate change in aortic architecture with reduced elastic fiber fragmentation (Fig. 7D). These observations indicate that the lack of myeloid Tsc2 could aggravate AngII-induced AAs formation by augmenting Gpr68-mediated macrophage inflammatory response and macrophage-derived Mmp9 expression.

## Discussion

Aortic aneurysm is a severe cardiovascular disease with a high risk of mortality related to rupture. There is an urgent need to discover effective pharmacological agents to decelerate or reverse the progression of AAs. The present study demonstrates that myeloid Tsc2 defciency aggravates AAs formation in vivo and that Gpr68 inhibition limits aneurysm enlargement via regulation of CREB phosphorylation, indicating that pharmacological modulation of Gpr68 may be a feasible approach for the treatment of AAs.

One major finding of this study is that Tsc2 deletion in macrophages exacerbated aortic aneurysms formation. It is interesting to observe that Tsc2 deletion markedly increased the the maximal descending thoracic and abdominal aortic diameter. Our study is in line with a previous study which showed that mutations in the Tsc2 gene result in descending aortic aneurysm. Although TAA and AAA have different aetiologies and pathogeneses, accumulating evidence supports the important role of a chronic inflammatory process in both TAA and AAA development [2]. Macrophages, the most abundant immune cells in the aneurysmal arterial wall, are critical mediators of aneurysm evocation and progression. Tsc2 deletion in myeloid cells induced increases in inflammatory response, macrophage infiltration and Mmp9 expression, which subsequently contribute to rupture of elastin fibers and aortic wall dilation. Tsc2 deletion induced pro-aneurysmal phenotype was consistent with the clinical studies that patient with Tsc2 mutation lead to aortic aneurysms formation [10, 27]. Our RNAseq data showed that Tsc2 deficient macrophages upregulated the expressions of inflammatory genes (including IL-6, IL-1β, MCP-1, Ccl7, Ccl12) and MMP genes (Mmp9, Mmp10, Mmp13) in the presence of AngII. Actually, mixed findings have been reported on the role of genetic manipulation of the Tsc1/Tsc2 in the production of inflammatory and immunomodulatory cytokines. For example, Pan et al [28]. demonstrated that Tsc1 deficient macrophages promotes the expression of proinflammatory cytokines in response to TLR stimulation due to increased activation of mTORC1. Moreover, Tsc1 knockout bone marrow-derived macrophages treated by lipopolysaccharide secreted more of the pro-inflammatory cytokines TNF-α and IL-6 but less of the anti-inflammatory cytokine IL-10 [29]. Zhu et al [12]. reported that Tsc1/2 complex inhibited M1 polarization in macrophages and that myeloid cell-specific Tsc1 knockout mice spontaneously developed an inflammatory disorder. Using Tsc2-deficient cells, including human angiomyolipoma parental cells and mouse embryonic fibroblasts (MEFs), Wang et al [30]. found that deletion of Tsc2 up-regulated IL-6 transcription and secretion compared to control cells. However, a research group reported that Tsc2 deficiency diminished the production of pro-inflammatory cytokines in MEFs and human monocytes [31]. The reasons for this inconsistency possibly due to the different cell types, context-dependent actions of macrophage Tsc1/2 and duration of Tsc1/2 inhibition/deletion used in these studies. This would require further clarification in future research.

Another major finding of this study is that elevated expression of Gpr68 in macrophages is responsible for the exacerbated aneurysms formation. RNA-Seq analysis identified Gpr68 (also known as Ogr1) as the most up-regulated gene in Tsc2 deficient macrophages. We also found elevated expression of Gpr68, particularly in macrophages from myeloid Tsc2 deficient aortas. Intriguingly, Gpr68 inhibition significantly suppressed the expression of proinflammatory cytokines and Mmp9 activity in Tsc2 deficient macrophages. In vivo, Gpr68 inhibition resulted in a marked reduction in AAs incidence and diameter. Gpr68 is activated by the extracellular acidic pH. Nevertheless, high-Gpr68-expressing macrophages were also found in inflammatory site and exerted pro-inflammatory effects in vivo and in vitro [17]. A database inquiry indicated that Gpr68 was mainly expressed in immune cells, including T cells and macrophages (Supplementary Fig. 2A). The expression of Gpr68 was significantly increased in lipopolysaccharide-induced pro-inflammatory macrophages (Supplementary Fig. 2A). High-Gpr68-expressing monocytes released more TNFα and IL-6 in response to lipopolysaccharide stimuli under natural conditions, and the lipopolysaccharide stimulated release of inflammatory cytokines was further increased under acidic conditions [17]. Moreover, Gpr68 inhibition in monocytes alleviates CKD induced cardiac inflammation [17]. Gpr68 deficient mice exhibit less severe airway inflammation in the ovalbumin-induced experimental asthma model [32]. These observations highlighted the importance of manipulation of Gpr68 in the regulation of macrophage-mediated inflammation.

More interestingly, glycolytic inhibitor 2-DG treatment partially attenuated the level of Gpr68, indicating that Tsc2 deficiency negatively regulates Gpr68 expression involving activation of glycolysis. Recent studies demonstrated a link between inflammation and glycolysis in the pathogenesis of aortic aneurysm [33, 34]. Enhanced glycolytic activity in aortic wall as well as in macrophages contributes to the pathogenesis of aneurysm development [35]. Tsc2 has been reported to mediate glycolysis in several diseases [5, 36]. The end point product of glycolysis is lactate that leads to extracellular acidosis. Under ischemic and inflammatory circumstances, such as ischaemic heart disease, atherosclerotic lesions, and rheumatoid arthritis, extracellular acidification occurs due to the stimulation of anaerobic glycolysis [37–39]. Using a microelectrode, Naghavi et al [38]. found pH values could be as low as 6.8 in the subendothelial areas of human carotid plaques. In addition, visualization of the atherosclerotic lesions with two pH-sensitive fluorescent dyes indicated that the pH may reach values even below 6 [38]. Importantly, a pH-sensitive probe pHrodo together with fluorescence reflectance imaging identified acidic regions at positions of macrophage accumulation in human and mouse abdominal aortic aneurysm lesions [40]. Gpr68-family GPCRs sense neutral or weak pH 6–8 through histidine residues [41]. Therefore, we speculate that Tsc2 deficiency-induced glycolysis and subsequent extracellular acidic pH mediates, at least partly, the upregulation of Gpr68 expression in the aorta of mice. On the basis of our present results, it will be an interesting future issue to measure pH in aneurysm tissues. Gpr68 is also expressed in endothelial cells of mammalian small-diameter blood vessels, which sense shear stress [42]. The high-Gpr68-expressing macrophages that infiltrated into the aorta may have been exposed to shear stress around vessels and were activated to produce inflammatory cytokines.

**Fig. 8:**
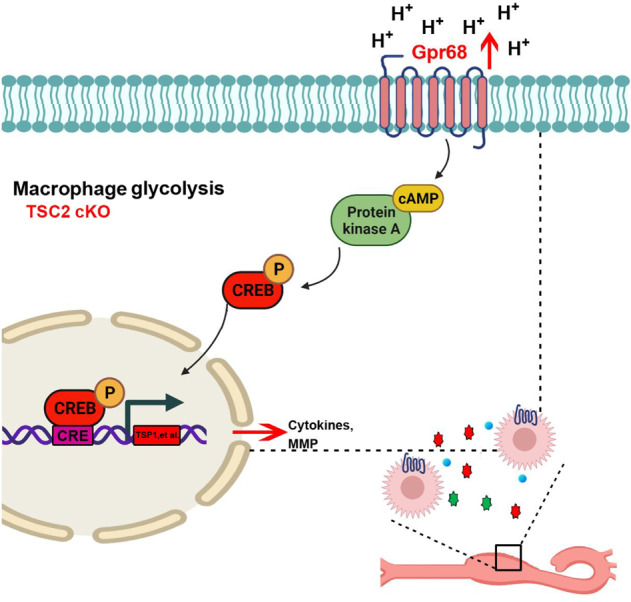
Working schematic of how Tsc2 depletion in macrophage aggravated AngII-induced aortic aneurysms formation. Tsc2 depletion enhanced glycolytic activity and upregulated Gpr68 expression in macrophages, resulting in increased transcription activity of CREB. CREB acts on TSP-1, Mmp9 and FOS, aggravating the inflammatory response and elastin degradation, further promoting the formation of AAs.

The protective effect of Gpr68 blockade might be due to modulation of phosphorylated CREB. Recent study by Cui et al [22]. revealed that the levels of phosphorylated CREB (p-CREB) in macrophages were increased significantly in AAD and phosphorylation of CREB by p38α promotes inflammatory reactions of macrophages. Additionally, CREB promotes transactivation of Fos gene family (Fosb, Fosl1, c-Fos) to enhance macrophage-mediated inflammation [24]. We observed that Gpr68 inhibition reversed the elevated level of phosphorylated CREB induced by lacking Tsc2 in macrophages. Of interest, increased expression of multiple genes in Tsc2 deficient macrophages including Mmp9, Fosl1, Fos and TSP-1 were involved as the downstream targets of CREB and associated with aneurysms formation. Therefore, Tsc2 may regulate inflammatory and proteolytic genes through Gpr68/CREB pathway. We developed a mechanistic model describing the role of macrophage Tsc2 in AAs formation (Fig. 8).

We acknowledge that there are limitations in our study. Lyz2-cre leads to deletion of Tsc2 in monocyte/macrophages, granulocytes, and few CD11c + dendritic cells [43]. Tsc2 may affect neutrophil function and thus contribute to aneurysm formation. Macrophage-targeted delivery vectors (including nano-delivery strategy) instead of shRNA lentiviral particles may be helpful for the treatment of aortic aneurysm in future study. The current study is also limited by the animal model. There is still no single animal model that could reflect the full disease spectrum of human aortic aneurysms. Further conditional Tsc2 knockout mice in other vascular cell types, as well as aortic aneurysms models will be more informative.

In summary, our data demonstrate that Tsc2 deletion triggers a proinflammatory phenotype in macrophages through the Gpr68/CREB pathway, thereby aggravating aortic remodeling and aortic aneurysm formation. Targeting macrophage Gpr68 may represent a novel potential therapeutic approach to limit aortic aneurysm expansion. Therefore, small molecule agents could be designed to block macrophage Gpr68 signaling to prevent aortic aneurysm development especially in patient with Tsc2 mutation.

## Materials and methods

### Mice and reagents

Tsc2 floxed mice were obtained from the Jackson Laboratory (JAX stock #027458). Lyz2-cre transgenic mice were kindly provided by Dr. Wen-cheng Zhang (Shandong University). Myeloid-specific Tsc2 deficient mice in an ApoE^–/–^ background were generated by crossing Tsc2 floxed mice (Tsc2^fl/fl^) with Lyz2-cre transgenic mice, and then crossing with ApoE^–/–^ mice to form Tsc2^flox/flox^ Lyz2-cre^+^ApoE^–/–^ mice, hereafter referred to as Tsc2^MKO^ApoE^–/–^ mice. Experiments were performed using sixteen-week-old male Tsc2^MKO^ApoE^–/–^ and Tsc2^flox/flox^Lyz2-cre^-^ (Tsc2^WT^)ApoE^–/–^ littermates. Genotyping was performed by PCR analysis according to The Jackson Laboratory’s protocol. For Gpr68 inhibition, shRNA lentiviral particles carrying Gpr68 gene silencing sequence (5ʹ-UAACAAUUCAGGUUCCUCGCCCUGG-3ʹ) or negative control (sh-NC) were synthesized as previously described [44] and purchased from BoShang Bio-tech Company (Jinan, China). sh-Gpr68 or sh-NC (2 × 10^7^ TU per mouse) was injected into the mice through the tail vein before AngII infusion. Randomization and allocation concealment were performed. All experimental protocols conformed to the Guide for the Care and Use of Laboratory Animals published by the US National Institutes of Health and were approved by the animal ethics committee of Shandong University.

Angiotensin II (AngII) were from Sigma Aldrich (St. Louis, MO, USA). Primary antibodies used include: Gpr68 (Cat#NBP2-93067, Cat#NLS1195) was purchased from Novus Biologicals (Littleton, USA). Tsc2 (Cat#4308 S), CREB (Cat#9197 S), phosphorylated CREB (Cat#9198 S), vimentin (Cat#5741 S), IL-6 (Cat#12912 S), MCP-1 (Cat#41987 S) and β-actin (Cat#3700 S) were purchased from Cell Signaling Technology (Danvers, USA). Mmp9 (Cat#ab38898), α-SMA (Cat#ab5694), sm22α (Cat#ab14106) and IL-1β (Cat#ab234437) were purchased from Abcam (Cambridge, UK). F4/80 (Cat#MCA497) were purchased from BioRad (Hercules, USA). Secondary antibodies used for western blot were from ZSGB-BIO (Beijing, China). Secondary antibodies used for immunofluorescence staining were purchased from Abcam (Cambridge, UK).

### Determination of blood pressure

Systolic blood pressure (SBP) was measured noninvasively on conscious mice using a noninvasive tail-cuff system (Natsume, Tokyo, Japan). Measurements were recorded at the same time of day throughout the study. Systolic blood pressure was measured at least five times, and the mean was used for each mice.

### Angiotensin II infusion

To induce aortic aneurysms formation, all mice received angiotensin II (1000 ng/kg/min) or saline by micro-osmotic pumps (Alzet model 2004; Durect, Cupertino, CA, USA) as we previously described [45]. All mice received regular laboratory chow diet throughout the study. Mice that died immediately after the osmotic pumps implantation were excluded from the present study. At 28 days after the osmotic pumps implantation, mouse aortas were harvested, and aneurysms were quantified. We measured the diameter of the ascending, descending thoracic, and abdominal aortic segments of the extracted aortas. An aneurysm was defined as ≥50% enlargement of the external diameter compared to aortas from saline-infused control mice. Necropsy was performed to confirm aortic rupture during AngII infusion. Operators responsible for the experimental procedures and data analysis were blinded to group allocation. The number of animals that received AngII infusion was determined based on prior experimentation in our lab.

### Histology and immunohistochemistry

Paraffin-embedded aortic sections (5 μm thick) were subjected to hematoxylin and eosin (H&E) and Verhoeff-Van Gieson Elastin staining (Sigma-Aldrich) for morphological assessment and detection of elastic fiber integrity, respectively. For immunohistochemical staining, sections were incubated with primary antibodies overnight at 4 °C, followed by secondary antibody before visualization with diaminobenzidine (Gene Tech, Shanghai, China). In the double immunofluorescence analysis, appropriate secondary antibodies (Alexa Fluor 488 and 594) were employed. DAPI was used for nuclei staining. Images were observed with a fluorescence microscope (Olympus, Tokyo, Japan).

### Cell cultures

Three days before collecting peritoneal macrophages, mice were intraperitoneally injected with 1 mL of 3.0% thioglycollate medium. Cells were further perfused from the peritoneal cavity and then cultured in RPMI 1640 medium containing 10% fetal bovine serum (FBS).

### Assays of oxygen consumption rate and extracellular acidification rate

Peritoneal macrophages were seeded at 4×10^4^ cells per well in Seahorse XF 96-well culture microplates. The cellular oxygen consumption rate (OCR) and extracellular acidification rate (ECAR) were assessed using the Seahorse XFe 96 Extracellular Flux Analyzer (Agilent Technologies, CA, USA) according to the manufacturer’s instructions. OCR and ECAR were determined using Seahorse XF Cell Mito Stress Test Kit and Seahorse XFe Glycolysis Stress Test Kit, respectively. Results were collected with Wave software (Agilent Technologies, CA, USA). OCR is indicated in pmol/minute and ECAR in mpH/minute.

### Transwell migration assay

Bone marrow-derived macrophages (BMDMs) were isolated from long bones and washed with PBS. Cells were starved 24 h prior to assay set up. 1×10^4^ cells/200 μl were suspended in medium without FBS and seeded into the upper chamber of transwell plates (Corning, USA) with 8 μm pore filters. The lower chamber was added with complete medium (containing 10% FBS). Following 12 h incubation at 37 °C, cells that migrated to the lower surface were stained with crystal violet. Migrated cells per field was quantified under a microscope.

### Real-time polymerase chain reaction (RT-PCR)

Total RNAs were extracted using TRIzol reagent (Invitrogen) and reverse-transcribed into cDNA using the PrimeScript reverse transcriptase reagent kit (Takara, Japan). All samples were amplified using SYBR Green-based RT-PCR. The mRNA levels were normalized to those of β-actin. Primer sequences for real-time quantification are listed in Table S4.

### Western blot assay

Total proteins were extracted from cultured cells or tissues with RIPA buffer. Proteins were separated by 10% SDS-PAGE and transferred to PVDF membranes (Millipore). Membranes were incubated with specific antibodies. After further incubation with horseradish peroxidase-conjugated secondary antibodies, bound primary antibodies were detected by chemiluminescence (Millipore, USA). The intensity of bands was quantified with ImageJ software (NIH, Bethesda, MD, USA).

### Gelatin zymography

Mmp9 activities were determined by a Mmp gelatin zymography kit (GenMed Scientific Inc., USA) according to the manufacturer’s instructions. In brief, the supernatant of macrophages culture medium was collected and mixed with sample buffer dye then separated by 10% SDS-PAGE gels containing 0.1% gelatin. The gels were further washed with renaturing buffer and incubated with zymography reaction buffer. Finally, the gels were stained with Coomassie Brilliant Blue.

### Microarray gene expression analysis

Total RNA was extracted from peritoneal macrophages in AngII-infused Tsc2^MKO^ApoE^−/−^ and Tsc2^WT^ApoE^−/−^ mice. The RNA concentration and integrity were determined by Agilent 2100 RNA nano 6000 assay kit (Agilent Technologies, CA, USA). The transcriptome sequencing experiments were performed by BoShang Bio-tech Company (Jinan, China). The differentially expressed genes were identified with a fold-change of >2.0 and *P* value < 0.05 between two groups.

### Statistical analysis

Statistical analysis was performed using SPSS16.0 software. Continuous variables were expressed as mean ± SD if normally distributed or median if non-normally distributed. Categorical data were expressed as frequency and percentage. We tested the normality and equal variance using the Kolmogorov-Smirnov test implemented in Prism statistical software (GraphPad, San Diego) before parametric data analysis. Comparison of 2 groups of normally distributed samples was done using unpaired Student’s *t* tests. Comparison of multiple groups of normally distributed samples was done using ANOVA, and Tukey was performed as a post test. Nonnormally distributed data were analyzed with Mann–Whitney test or the Kruskal-Wallis test, as appropriate. Categorical data were analyzed by chi-square test or Fisher’s exact test. *P* < 0.05 was considered statistically significant.

## Supplementary information

Supplemental Figure and Table

Full and uncropped western blots

A reproducibility checklist

## Data Availability

The experimental data sets generated and/or analyzed during the current study are available from the corresponding author upon reasonable request.
