# Supplementary material for: Loss of myeloid Tsc2 predisposes to angiotensin II-induced aortic aneurysm formation in mice
Source: Cell Death Dis. 2022 Nov 18;13(11):972. doi: 10.1038/s41419-022-05423-2 (PMC9674579; doi:10.1038/s41419-022-05423-2)
Supplement: Supplementary file 1 — Supplemental Figure and Table [file 41419_2022_5423_MOESM1_ESM.pdf]

Supplemental Figure

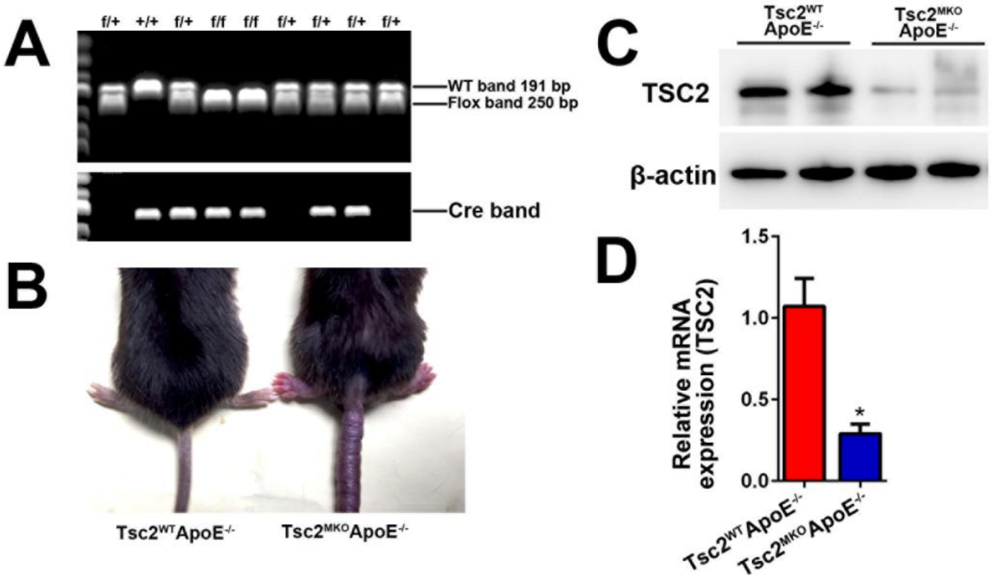

Fig. S1. Phenotypic evaluation of myeloid Tsc2-deficient mice. (A) PCR validation of WT, Tsc2$^{f/+}$, and Tsc2$^{f/f}$ alleles (Upper) and Cre recombinase transgene (Lower). (B) Image of Tsc2^{MKO}ApoE$^{-/-}$ and control mice at the age of 6 months. (C and D) Expression levels of Tsc2 in the macrophages of Tsc2^{MKO}ApoE$^{-/-}$ and control mice determined by western blot (n=4) and RT-PCR (n=5). *P<0.05, vs. Tsc2^{WT}ApoE$^{-/-}$ mice.

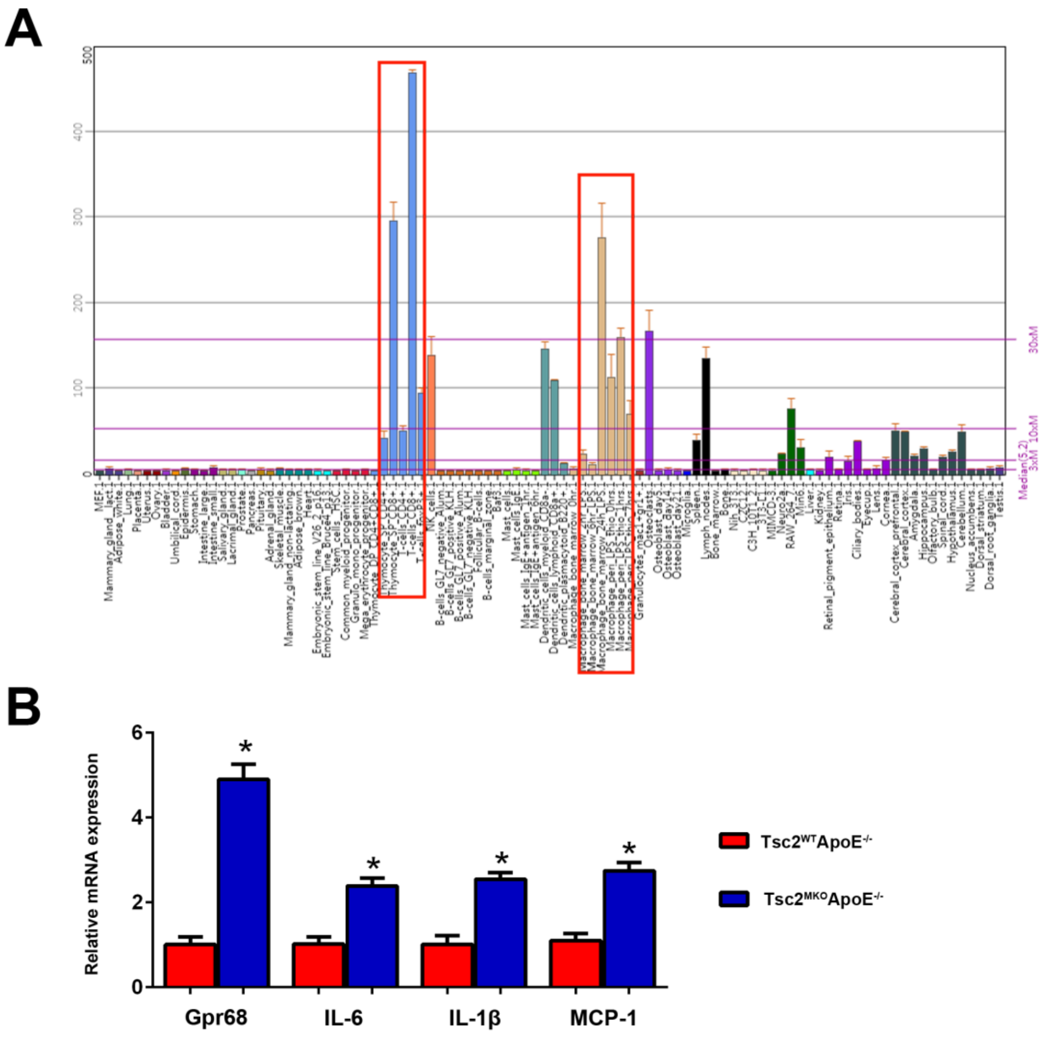

Fig. S2. (A) Gpr68 search on BioGPS (http://biogps.org/). Gpr68 was mainly expressed in immune cells, including T cells and macrophages. The expression of Gpr68 was significantly increased in lipopolysaccharide-induced pro-inflammatory macrophages. (B) Myeloid-specific deletion of Tsc2 increases the expression of Gpr68 as well as proinflammatory genes in aortic $CD11b^{+}$ myeloid cells from AngII-infused $Tsc2^{MKO}ApoE^{-/-}$ and $Tsc2^{WT}ApoE^{-/-}$ mice (n=5). *P<0.05, vs. $Tsc2^{WT}ApoE^{-/-}$ mice infused with AngII for 28 d.

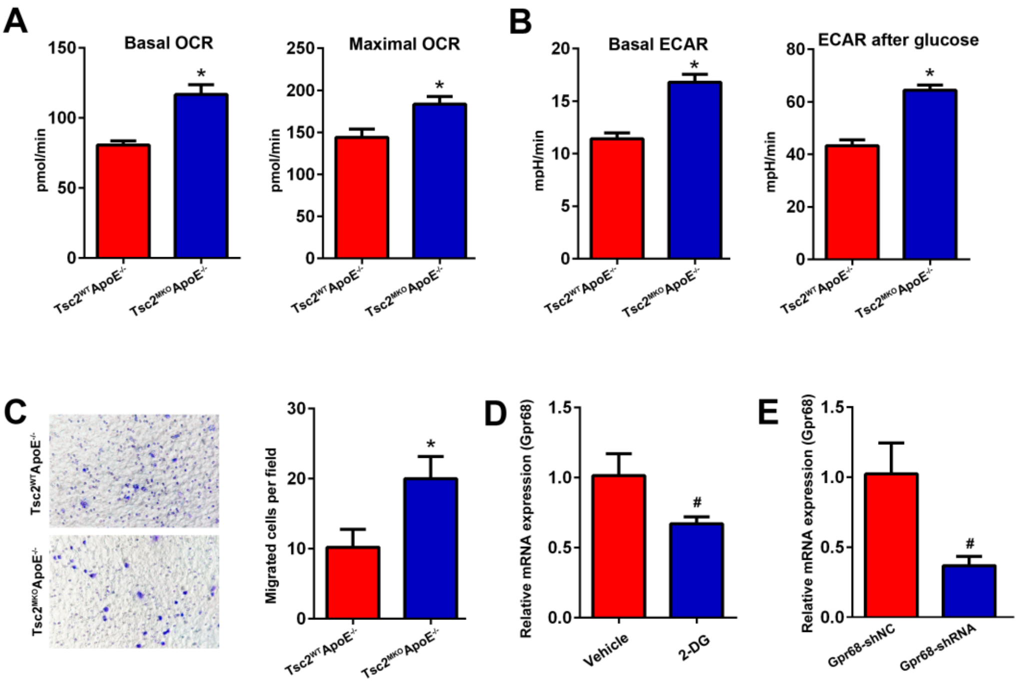

Fig. S3. (A) Seahorse analysis of basal and maximal oxygen consumption rate (OCR) in the peritoneal macrophages from Tsc2^{MKO}ApoE$^{-/-}$ and Tsc2^{WT}ApoE$^{-/-}$ infused with AngII for 7 d (n=5). (B) Seahorse analysis of basal extracellular acidification rates (ECAR) and ECAR after glucose injection in the peritoneal macrophages from Tsc2^{MKO}ApoE$^{-/-}$ and Tsc2^{WT}ApoE$^{-/-}$ infused with AngII for 7 d (n=5). (C) Tsc2 deficient bone marrow-derived macrophages showed signifcantly increased migration (n=5). (D) Macrophages from Ang II-infused Tsc2^{MKO}ApoE$^{-/-}$ mice were treated with 2-deoxy-D-glucose (2-DG, 2 mg/mL) or vehicle for 24 hours. Quantitative real-time PCR analysis of Gpr68 mRNA expression (n=5). (E) Macrophages from Ang II-infused Tsc2^{MKO}ApoE$^{-/-}$ mice were transfected with shRNA-Gpr68 or negative control shRNA for 24 hours. Quantitative real-time PCR analysis of Gpr68 mRNA expression (n=5). **P*<0.05, vs. Tsc2^{WT}ApoE$^{-/-}$ mice infused with AngII. $^{\#}$*P*<0.05, vs. Vehicle or Gpr68-shNC group.

Supplemental Table

Table S1. Blood pressure and body weight in AngII-infused mice

	Saline		Ang II		Ang II-infused $Tsc2^{MKO}ApoE^{-/-}$	
	$Tsc2^{WT}ApoE^{-/-}$	$Tsc2^{MKO}ApoE^{-/-}$	$Tsc2^{WT}ApoE^{-/-}$	$Tsc2^{MKO}ApoE^{-/-}$	Gpr68-shNC	Gpr68-shRNA
Body weight 4W(g)	27.95 ±0.95	27.69 ±0.65	28.39 ±0.53	28.10 ±0.71	28.03 ±0.60	27.46 ±0.69
SBP 4W(mmHg)	102.9 ±4.5	105.8 ±5.7	147.0 ±7.5*	152.5 ±7.7*	151.9 ±7.8	148.4 ±5.8

N = 8 in each group. *P < 0.05 compared to same genotypic mice with saline infusion.

Table S2. AAs formation in AngII-infused mice

Groups	Total number	AAA(Abdominal aortic rupture/AAA without rupture)	TAA(Thoracic aortic rupture/TAA without rupture)
Saline-infused $Tsc2^{WT}ApoE^{-/-}$	10	0 (0/0)	0 (0/0)
Saline-infused $Tsc2^{MKO}ApoE^{-/-}$	10	0 (0/0)	0 (0/0)
AngII-infused $Tsc2^{WT}ApoE^{-/-}$	17	8 (1/7)	2 (0/2)
AngII-infused $Tsc2^{MKO}ApoE^{-/-}$	18	15 (5/10)	10 (2/8)
AngII-infused $Tsc2^{MKO}ApoE^{-/-}$+ Gpr68-shNC	15	12 (4/8)	8 (2/6)
AngII-infused $Tsc2^{MKO}ApoE^{-/-}$+ Gpr68-shRNA	15	4 (1/3)	1 (0/1)

Table S3. Detailed list of differentially expressed genes with over 2-fold upregulation or over 2-fold downregulation in Tsc2-deficient macrophages.

Gene	Log2FoldChange	pval	padj	Up/ Down	GeneName	Position
ENSMUSG00000047415	7.049699145	1.06E-31	5.79E-28	up	Gpr68	chr12:100876682-100908198:-
ENSMUSG00000003657	4.888674667	0.018383833	0.99977258	up	Calb2	chr8:110137502-110168210:-
ENSMUSG00000040752	4.861449495	0.026432407	0.99977258	up	Myh6	chr14:54941921-54966927:-
ENSMUSG00000063851	4.815059543	0.011131856	0.880814703	up	Rnf183	chr4:62427540-62435252:-
ENSMUSG00000026090	4.503807328	0.02318757	0.99977258	up	2010300C02Rik	chr1:37611677-37720085:-
ENSMUSG00000051243	4.46059206	0.025265416	0.99977258	up	Islr2	chr9:58196297-58204319:-
ENSMUSG00000024803	4.300398039	5.78E-05	0.067702424	up	Ankrd1	chr19:36111965-36119844:-
ENSMUSG00000092035	3.975957396	0.028749441	0.99977258	up	Peg10	chr6:4747306-4760517:+
ENSMUSG00000079164	3.955328297	0.022062757	0.99977258	up	Tlr5	chr1:182954788-182976044:+
ENSMUSG00000022304	3.923681046	0.026346184	0.99977258	up	Dpys	chr15:39768487-39857470:-
ENSMUSG00000102323	3.881120523	0.031554703	0.99977258	up	Gm37790	chr2:156846370-156849715:-
ENSMUSG00000029378	3.843962778	0.033856083	0.99977258	up	Areg	chr5:91139599-91148432:+
ENSMUSG00000025993	3.700973531	6.45E-06	0.009621364	up	Slc40a1	chr1:45908068-45926523:-
ENSMUSG00000026442	3.655138448	0.048169057	0.99977258	up	Nfasc	chr1:132564690-132741797:-
ENSMUSG00000047562	3.629213782	0.00015764	0.129264621	up	Mmp10	chr9:7502352-7510240:+
ENSMUSG00000046203	3.561237819	0.018086497	0.99977258	up	Sprr2g	chr3:92373915-92375229:+
ENSMUSG00000091345	3.289365747	0.033009928	0.99977258	up	Col6a5	chr9:105856078-105960643:-
ENSMUSG00000027456	3.165002263	0.005396606	0.678728727	up	Sdcbp2	chr2:151572622-151590005:+
ENSMUSG00000074634	2.911610337	0.029593669	0.99977258	up	Tmem267	chr13:119488039-119611059:+
ENSMUSG00000017412	2.810260969	9.18E-05	0.088352524	up	Cacnb4	chr2:52428320-52676831:-
ENSMUSG00000093765	2.794354092	0.000245469	0.167736866	up	Gm20658	chr12:116102769-116138648:-
ENSMUSG00000026072	2.791847671	3.49E-07	0.000715454	up	Il1r1	chr1:40225080-40317257:+
ENSMUSG00000069515	2.7828123	8.63E-07	0.001572047	up	Lyz1	chr10:117287797-117292868:-
ENSMUSG00000040152	2.770369248	0.035318847	0.99977258	up	Thbs1	chr2:118111876-118127133:+
ENSMUSG00000060509	2.760170165	0.016861618	0.993012722	up	Xcr1	chr9:123852315-123862029:-
ENSMUSG00000028358	2.703692853	0.000230717	0.166954246	up	Zfp618	chr4:62965573-63139708:+
ENSMUSG00000024112	2.679979753	0.036825503	0.99977258	up	Cacna1h	chr17:25374285-25433783:-
ENSMUSG00000027022	2.621918077	0.013551397	0.928172059	up	Xirp2	chr2:67446002-67526614:+
ENSMUSG00000028716	2.615229402	0.019022151	0.99977258	up	Pdzk1ip1	chr4:115088708-115093899:+
ENSMUSG00000098534	2.587547953	0.026696518	0.99977258	up	Gm27167	chr8:86902453-86919422:+
ENSMUSG00000026815	2.484055031	0.027915108	0.99977258	up	Gfi1b	chr2:28609450-28621982:-
ENSMUSG00000016349	2.483226126	0.043730564	0.99977258	up	Eef1a2	chr2:181147653-181157014:-
ENSMUSG00000028194	2.460610396	0.004626997	0.632356235	up	Ddah1	chr3:145758675-145894277:+
ENSMUSG00000074934	2.458264222	0.003036889	0.549868097	up	Grem1	chr2:113746164-113758646:-
ENSMUSG00000097585	2.455164682	0.001594945	0.409062651	up	E230029C05Rik	chr7:89980723-90049069:+

ENSMUSG00000032899	2.342917199	0.002190682	0.454774515	up	Styk1	chr6:131299142-131353597:-
ENSMUSG00000011267	2.339618036	0.048346732	0.99977258	up	Zfp296	chr7:19577287-19580656:+
ENSMUSG00000027398	2.33485094	1.39E-08	5.70E-05	up	Il1b	chr2:129364570-129371139:-
ENSMUSG00000026725	2.327085271	0.037865493	0.99977258	up	Tnn	chr1:160085029-160153580:-
ENSMUSG00000021411	2.301117345	0.045704371	0.99977258	up	Pxdc1	chr13:34627834-34653486:-
ENSMUSG00000054793	2.289384669	0.037061687	0.99977258	up	Cadm4	chr7:24482023-24504539:+
ENSMUSG00000033965	2.270578501	0.001266089	0.384515992	up	Slc16a2	chrX:103697414-103821983:-
ENSMUSG00000046259	2.177009973	0.010070093	0.848802638	up	Sprr2h	chr3:92385685-92387324:+
ENSMUSG00000057604	2.168271492	0.030654869	0.99977258	up	Lmcd1	chr6:112273758-112330425:+
ENSMUSG00000086755	2.156795913	0.00881134	0.823582002	up	Gm11216	chr4:64075533-64083814:+
ENSMUSG00000087203	2.149887225	0.019961793	0.99977258	up	Gm13986	chr2:117857291-118111202:-
ENSMUSG00000032332	2.142596595	0.012989934	0.928172059	up	Col12a1	chr9:79598991-79718831:-
ENSMUSG00000052353	2.142300556	0.041544516	0.99977258	up	Cemip	chr7:83932857-84086502:-
ENSMUSG00000028459	2.134202822	8.64E-06	0.011804773	up	Cd72	chr4:43446462-43454628:-
ENSMUSG00000029093	2.127663031	0.015449393	0.967242849	up	Sorcs2	chr5:36017180-36398139:-
ENSMUSG00000044103	2.123891708	0.000485329	0.227411311	up	Il1f9	chr2:24186476-24193568:+
ENSMUSG00000026700	2.120201679	0.004315254	0.617836897	up	Tnfsf4	chr1:161395409-161418410:+
ENSMUSG00000028364	2.091162788	0.000434137	0.227219987	up	Tnc	chr4:63959785-64047015:-
ENSMUSG00000022501	2.066650678	0.011751257	0.884039485	up	Prm1	chr16:10796326-10796886:-
ENSMUSG00000006403	2.054729254	0.029516772	0.99977258	up	Adamts4	chr1:171250421-171260637:+
ENSMUSG00000103367	2.049026964	0.014971346	0.966653809	up	Gm38158	chr1:176835724-176836062:+
ENSMUSG00000044716	2.039654987	0.001435126	0.398190151	up	Dok7	chr5:35056766-35087839:+
ENSMUSG00000052942	2.036093122	0.000974572	0.332978812	up	Glis3	chr19:28258851-28680077:-
ENSMUSG00000027859	2.01490265	0.002410546	0.479468024	up	Ngf	chr3:102469919-102521013:+
ENSMUSG00000035357	1.98680694	0.025216847	0.99977258	up	Pdzrn3	chr6:101149609-101377897:-
ENSMUSG00000081058	1.982252524	0.015800186	0.97049834	up	Hist2h3c2	chr3:96238108-96239127:-
ENSMUSG00000018916	1.978767692	0.027446584	0.99977258	up	Csf2	chr11:54247271-54249667:-
ENSMUSG00000060183	1.973424513	0.000442195	0.227219987	up	Cxcl11	chr5:92359544-92365485:-
ENSMUSG00000097164	1.972163476	0.000276982	0.176574875	up	Cep83os	chr10:94671025-94688576:-
ENSMUSG00000032373	1.964480949	0.028436257	0.99977258	up	Car12	chr9:66713686-66766845:+
ENSMUSG00000002289	1.956306359	0.006747469	0.718561609	up	Angptl4	chr17:33773750-33781575:-
ENSMUSG00000046733	1.944681539	0.023667073	0.99977258	up	Gprc5a	chr6:135065651-135084709:+
ENSMUSG00000048332	1.917917124	0.019665859	0.99977258	up	Lhfp	chr3:53041528-53261679:+
ENSMUSG00000010830	1.90341489	0.033396903	0.99977258	up	Kdelr3	chr15:79516408-79527739:+
ENSMUSG00000038400	1.896427025	0.001640907	0.409062651	up	Pmepa1	chr2:173224458-173276533:-
ENSMUSG00000035914	1.875732488	0.004723116	0.640157831	up	Cd276	chr9:58524298-58555437:-
ENSMUSG00000022037	1.861375604	0.003095073	0.549868097	up	Clu	chr14:65968483-65981547:+
ENSMUSG00000042367	1.852136206	0.031112206	0.99977258	up	Gjb3	chr4:127325235-127330844:-
ENSMUSG00000079339	1.838829001	0.000573068	0.259170028	up	Ifit1bl1	chr19:34592888-34601968:-
ENSMUSG00000037995	1.820062466	0.019665785	0.99977258	up	Igsf9	chr1:172481788-172498878:+
ENSMUSG00000009281	1.810706957	0.033704844	0.99977258	up	Rarres2	chr6:48569696-48572789:-
ENSMUSG00000066975	1.808564971	0.018565882	0.99977258	up	Cryba4	chr5:112246493-112252518:-
ENSMUSG00000020205	1.799662758	0.013137846	0.928172059	up	Phlda1	chr10:111506286-111508645:

						+
ENSMUSG00000044337	1.794018711	0.031974006	0.99977258	up	Ackr3	chr1:90203980-90216751:+
ENSMUSG00000071984	1.785157001	0.038648176	0.99977258	up	Fndc1	chr17:7738569-7827302:-
ENSMUSG00000028965	1.777998184	0.009005506	0.823582002	up	Tnfrsf9	chr4:150914562-150946102:+
ENSMUSG00000026204	1.770145675	0.005591588	0.679274355	up	Ptprn	chr1:75247027-75264502:-
ENSMUSG00000040133	1.755475936	0.023342948	0.99977258	up	Gpr176	chr2:118277110-118373419:-
ENSMUSG00000020614	1.743866061	0.025021622	0.99977258	up	Fam20a	chr11:109669749-109722279:-
ENSMUSG00000026475	1.742933048	0.009767426	0.830928841	up	Rgs16	chr1:153740349-153745468:+
ENSMUSG00000027463	1.737562829	0.001416253	0.398190151	up	Slc52a3	chr2:151996511-152009258:+
ENSMUSG00000027408	1.736759155	0.022672046	0.99977258	up	Cpxm1	chr2:130390775-130397574:-
ENSMUSG00000031963	1.735168508	0.040313416	0.99977258	up	Bmper	chr9:23223076-23485200:+
ENSMUSG00000091243	1.728859874	0.004332393	0.617836897	up	Vgll3	chr16:65815257-65866368:+
ENSMUSG00000048376	1.724793136	0.048186294	0.99977258	up	F2r	chr13:95601803-95618487:-
ENSMUSG00000031955	1.722925053	0.004804822	0.64589417	up	Bcar1	chr8:111710474-111743809:-
ENSMUSG00000076431	1.715866369	0.014326165	0.943352482	up	Sox4	chr13:28948919-28953713:-
ENSMUSG00000045838	1.714301434	0.036726003	0.99977258	up	A430105I19Rik	chr2:118754158-118762661:-
ENSMUSG00000023341	1.708709345	0.001717014	0.414103495	up	Mx2	chr16:97535308-97560900:+
ENSMUSG00000038587	1.683906998	0.024624409	0.99977258	up	Akap12	chr10:4266380-4359470:+
ENSMUSG00000024041	1.677117775	0.04206603	0.99977258	up	Cryaa	chr17:31677933-31681724:+
ENSMUSG00000029371	1.674306454	0.003256643	0.550235128	up	Cxcl5	chr5:90759360-90761624:+
ENSMUSG00000046323	1.673740614	0.043096109	0.99977258	up	Dppa3	chr6:122626410-122630272:+
ENSMUSG00000032374	1.666129008	0.002979623	0.549868097	up	Plod2	chr9:92542223-92608428:+
ENSMUSG00000051029	1.658834748	0.023675241	0.99977258	up	Serpinb1b	chr13:33078575-33094380:+
ENSMUSG00000021707	1.657302064	0.000850233	0.306988867	up	Dhfr	chr13:92354726-92389053:+
ENSMUSG00000087256	1.65644646	0.015742532	0.97049834	up	Gm15990	chr10:86125827-86129854:+
ENSMUSG00000055976	1.649506737	0.021540855	0.99977258	up	Cldn23	chr8:35824712-35826559:-
ENSMUSG00000049608	1.648381952	0.004400959	0.622204514	up	Gpr55	chr1:85938318-85961007:-
ENSMUSG00000028128	1.640961639	0.001809203	0.423870513	up	F3	chr3:121723537-121735048:+
ENSMUSG00000009093	1.62707127	0.001174657	0.366715835	up	Gstt4	chr10:75814943-75822543:-
ENSMUSG00000069830	1.623563672	0.032477338	0.99977258	up	Nlrp1a	chr11:71092236-71144704:-
ENSMUSG00000005413	1.619108047	0.007778466	0.764473907	up	Hmox1	chr8:75093621-75100589:+
ENSMUSG00000020638	1.612284709	0.000861066	0.306988867	up	Cmpk2	chr12:26469204-26479837:+
ENSMUSG00000030782	1.583235061	0.047416302	0.99977258	up	Tgfb1i1	chr7:128246812-128255699:+
ENSMUSG00000090192	1.581947508	0.039654824	0.99977258	up	Gm16556	chr6:122560044-122570849:-
ENSMUSG00000015437	1.580733946	0.021986411	0.99977258	up	Gzmb	chr14:56258838-56262260:-
ENSMUSG00000030790	1.578667341	0.03483181	0.99977258	up	Adm	chr7:110627661-110629820:+
ENSMUSG00000024912	1.577908994	0.001608653	0.409062651	up	Fosl1	chr19:5447703-5455945:+
ENSMUSG00000019997	1.566299978	0.001749682	0.415866367	up	Ctgf	chr10:24595442-24598683:+
ENSMUSG00000001506	1.557553379	0.009717802	0.830928841	up	Col1a1	chr11:94936224-94953042:+
ENSMUSG00000025746	1.55436197	0.000787804	0.306988867	up	Il6	chr5:30013114-30019981:+
ENSMUSG00000053846	1.534139162	0.00344127	0.563071516	up	Lipg	chr18:74939322-74961263:-
ENSMUSG00000032875	1.531153118	0.017386637	0.994763824	up	Arhgef17	chr7:100869752-100932107:-
ENSMUSG00000049653	1.526325026	0.00205701	0.440552524	up	Spatc1	chr15:76268089-76292572:+

ENSMUSG00000037363	1.518467463	0.007270914	0.74754304	up	Letm2	chr8:25578490-25597582:-
ENSMUSG00000026355	1.515563558	0.000793817	0.306988867	up	Mcm6	chr1:128331590-128359664:-
ENSMUSG00000036545	1.513277911	0.033026887	0.99977258	up	Adamts2	chr11:50602084-50807573:+
ENSMUSG00000024304	1.505232168	0.042306601	0.99977258	up	Cdh2	chr18:16588877-16809246:-
ENSMUSG00000051379	1.504237551	0.005540536	0.678728727	up	Flrt3	chr2:140650914-140671469:-
ENSMUSG00000054675	1.496259659	0.005475423	0.678728727	up	Tmem119	chr5:113793729-113800516:-
ENSMUSG00000035352	1.492075685	0.007293103	0.74754304	up	Ccl12	chr11:82101845-82103400:+
ENSMUSG00000030022	1.48165738	0.036889945	0.99977258	up	Adamts9	chr6:92772699-92943492:-
ENSMUSG00000021185	1.48144009	0.000584713	0.259170028	up	Dglucy	chr12:100779057-100896981:+
ENSMUSG00000107111	1.470868684	0.049317668	0.99977258	up	Gm40304	chr5:53546351-53547322:-
ENSMUSG00000001768	1.4670067	0.006958693	0.726895275	up	Rin2	chr2:145784734-145887616:+
ENSMUSG00000042745	1.462041489	0.019768342	0.99977258	up	Id1	chr2:152736251-152737410:+
ENSMUSG00000055435	1.460747387	0.013110369	0.928172059	up	Maf	chr8:115682942-115707794:-
ENSMUSG00000027800	1.458806121	0.032091849	0.99977258	up	Tm4sf1	chr3:57285611-57301988:-
ENSMUSG00000035373	1.456479867	9.70E-05	0.088352524	up	Ccl7	chr11:82045712-82047525:+
ENSMUSG00000033644	1.453362505	0.02329565	0.99977258	up	Piwil2	chr14:70372477-70429383:-
ENSMUSG00000028195	1.452978862	0.007364471	0.749766732	up	Cyr61	chr3:145646976-145649981:-
ENSMUSG00000029664	1.452975196	0.046168787	0.99977258	up	Tfpi2	chr6:3962595-3988919:-
ENSMUSG00000026042	1.443976584	0.015229324	0.967242849	up	Col5a2	chr1:45374321-45503282:-
ENSMUSG00000022995	1.440934251	0.030667283	0.99977258	up	Enah	chr1:181896384-182019990:-
ENSMUSG00000112798	1.437168676	0.0425674	0.99977258	up	Gm40685	chr10:69312139-69315376:+
ENSMUSG00000034898	1.432971999	0.002503407	0.48876042	up	Filip1	chr9:79815051-80012851:-
ENSMUSG00000024190	1.432760405	0.002068448	0.440552524	up	Dusp1	chr17:26505590-26508519:-
ENSMUSG00000032035	1.422716381	0.002694972	0.513924932	up	Ets1	chr9:32636221-32757820:+
ENSMUSG00000002020	1.422102261	0.002332279	0.472214444	up	Ltbp2	chr12:84783212-84876532:-
ENSMUSG00000020623	1.418839246	0.038193285	0.99977258	up	Map2k6	chr11:110399122-110525522:+
ENSMUSG00000022114	1.417739417	0.01436951	0.943352482	up	Spry2	chr14:105891947-105896819:-
ENSMUSG00000050953	1.411529813	0.009284986	0.823582002	up	Gja1	chr10:56377330-56402513:+
ENSMUSG00000028972	1.408837325	0.041936984	0.99977258	up	Car6	chr4:150187015-150201332:-
ENSMUSG00000038463	1.402372032	0.027598712	0.99977258	up	Olfml2b	chr1:170644532-170682789:+
ENSMUSG00000048895	1.401347029	0.004597169	0.632356235	up	Cdk5r1	chr11:80477023-80481184:+
ENSMUSG00000041992	1.399382326	0.005409332	0.678728727	up	Rapgef5	chr12:117516479-117759737:+
ENSMUSG00000068699	1.390752095	0.009240608	0.823582002	up	Flnc	chr6:29433256-29461883:+
ENSMUSG00000031520	1.385323333	0.031101251	0.99977258	up	Vegfc	chr8:54077606-54187096:+
ENSMUSG00000020256	1.377857063	0.025581449	0.99977258	up	Aldh1l2	chr10:83487450-83534140:-
ENSMUSG00000038067	1.374574884	0.031741708	0.99977258	up	Csf3	chr11:98701263-98703629:+
ENSMUSG00000049103	1.36650499	0.045821053	0.99977258	up	Ccr2	chr9:124101950-124113557:+
ENSMUSG00000047669	1.365137354	0.022565183	0.99977258	up	Msl3l2	chr10:56106917-56116880:+
ENSMUSG00000002489	1.360394286	0.011652906	0.882417961	up	Tiam1	chr16:89787111-89980080:-
ENSMUSG00000024232	1.359081628	0.044984726	0.99977258	up	Bambi	chr18:3507957-3516404:+

ENSMUSG00000020330	1.358431339	0.005862025	0.688898751	up	Hmmr	chr11:40701395-40733422:-
ENSMUSG00000044017	1.3528485	0.020725012	0.99977258	up	Adgrd1	chr5:129096750-129204599:+
ENSMUSG00000033149	1.349778243	0.013641987	0.928334375	up	Phldb2	chr16:45746243-45953598:-
ENSMUSG00000027276	1.346291703	0.001025289	0.343158065	up	Jag1	chr2:137081456-137116644:-
ENSMUSG00000029661	1.344478226	0.013174145	0.928172059	up	Col1a2	chr6:4504814-4541544:+
ENSMUSG00000070436	1.344021143	0.005414766	0.678728727	up	Serpinh1	chr7:99345376-99353239:-
ENSMUSG00000037411	1.343628064	0.01859261	0.99977258	up	Serpine1	chr5:137061504-137072268:-
ENSMUSG00000022098	1.335507571	0.009390844	0.823582002	up	Bmp1	chr14:70474558-70520234:-
ENSMUSG00000001555	1.328177392	0.043111269	0.99977258	up	Fkbp10	chr11:100415697-100424824:<br>+
ENSMUSG00000031523	1.323436795	0.036900366	0.99977258	up	Dlc1	chr8:36567751-36953143:-
ENSMUSG00000082329	1.322274579	0.035286066	0.99977258	up	Gm14287	chr2:157528869-157528985:-
ENSMUSG00000020399	1.320659442	0.001940798	0.436014808	up	Havcr2	chr11:46454935-46481255:+
ENSMUSG00000043939	1.318299189	0.024402201	0.99977258	up	A530064D06Rik	chr17:48151896-48167270:-
ENSMUSG00000023349	1.315972056	0.021455211	0.99977258	up	Clec4n	chr6:123229843-123247021:+
ENSMUSG00000096140	1.309051922	0.010483423	0.863960495	up	Ankrd66	chr17:43534174-43543639:-
ENSMUSG00000027562	1.308674611	0.037907619	0.99977258	up	Car2	chr3:14886273-14900770:+
ENSMUSG00000019929	1.308615937	0.01719515	0.99313989	up	Dcn	chr10:97479609-97518143:+
ENSMUSG00000070524	1.303930532	0.000390642	0.227219987	up	Fcrlb	chr1:170907273-170912941:-
ENSMUSG00000031673	1.302648426	0.010678429	0.866961538	up	Cdh11	chr8:102632095-102785642:-
ENSMUSG00000027219	1.300020696	0.006050937	0.693675995	up	Slc28a2	chr2:122426477-122461137:+
ENSMUSG00000056290	1.293149931	0.02616726	0.99977258	up	Ms4a4b	chr19:11443553-11463549:+
ENSMUSG00000021250	1.27444439	0.012891456	0.928172059	up	Fos	chr12:85473890-85477273:+
ENSMUSG00000032501	1.273780229	0.037087788	0.99977258	up	Trib1	chr15:59648350-59657099:+
ENSMUSG00000026566	1.27282292	0.025107362	0.99977258	up	Mpzl1	chr1:165592240-165634538:-
ENSMUSG00000052688	1.272196006	0.001671171	0.409062651	up	Rab7b	chr1:131688695-131715439:+
ENSMUSG00000036330	1.271560279	0.01736853	0.994763824	up	Slc18a1	chr8:69037711-69089235:-
ENSMUSG00000030257	1.271392908	0.007602339	0.764473907	up	Srgap3	chr6:112717971-112947266:-
ENSMUSG00000021453	1.270412536	0.031990826	0.99977258	up	Gadd45g	chr13:51846678-51848468:+
ENSMUSG00000030745	1.268482771	0.005141722	0.674593974	up	Il21r	chr7:125603429-125633570:+
ENSMUSG00000020303	1.26044628	0.015541532	0.967242849	up	Stc2	chr11:31357307-31370074:-
ENSMUSG00000000386	1.260156011	0.005875142	0.688898751	up	Mx1	chr16:97447035-97462907:-
ENSMUSG00000031538	1.258880243	0.035952235	0.99977258	up	Plat	chr8:22757727-22782844:+
ENSMUSG00000050578	1.257210666	0.012499884	0.923414878	up	Mmp13	chr9:7272514-7283331:+
ENSMUSG00000018593	1.25685868	0.029007368	0.99977258	up	Sparc	chr11:55394500-55423183:-
ENSMUSG00000027737	1.251919219	0.024086863	0.99977258	up	Slc7a11	chr3:49892526-50443614:-
ENSMUSG00000031375	1.251634836	0.016953876	0.993012722	up	Bgn	chrX:73483602-73495933:+
ENSMUSG00000024924	1.250068585	0.036097287	0.99977258	up	Vldlr	chr19:27216484-27254231:+
ENSMUSG00000073557	1.242201552	0.011608263	0.882417961	up	Ppp1r12b	chr1:134754658-134955942:-
ENSMUSG00000092920	1.239276478	0.038751857	0.99977258	up	Mirt2	chr15:76267135-76267419:-
ENSMUSG00000038235	1.234857615	0.041056118	0.99977258	up	F11r	chr1:171437535-171464603:+
ENSMUSG00000056758	1.228517405	0.016902186	0.993012722	up	Hmga2	chr10:120361275-120476469:-
ENSMUSG00000078851	1.227904248	0.024411033	0.99977258	up	Hist3h2a	chr11:58954685-58956830:+

ENSMUSG00000085431	1.226223827	0.033604106	0.99977258	up	4930440I19Rik	chr2:78051177-78194430:+
ENSMUSG00000044786	1.224181306	0.033632563	0.99977258	up	Zfp36	chr7:28376784-28380253:-
ENSMUSG00000072941	1.223708252	0.009506725	0.829310047	up	Sod3	chr5:52363791-52371418:+
ENSMUSG00000029761	1.22288746	0.019264722	0.99977258	up	Cald1	chr6:34598500-34775473:+
ENSMUSG00000004891	1.216030166	0.019263563	0.99977258	up	Nes	chr3:87971078-87980451:+
ENSMUSG00000039911	1.21399118	0.01776852	0.99977258	up	Spsb1	chr4:149896283-149955043:-
ENSMUSG00000034485	1.211474006	0.034847877	0.99977258	up	Uaca	chr9:60794542-60880370:+
ENSMUSG00000042182	1.204584713	0.010479537	0.863960495	up	Bend6	chr1:33852052-33907816:-
ENSMUSG00000029096	1.204579232	0.02831675	0.99977258	up	Htra3	chr5:35652023-35679782:-
ENSMUSG00000036181	1.200473448	0.026125916	0.99977258	up	Hist1h1c	chr13:23738808-23740367:+
ENSMUSG00000018906	1.191961884	0.008425681	0.803378854	up	P4ha2	chr11:54100095-54131665:+
ENSMUSG00000112375	1.188689468	0.004262767	0.617836897	up	Gm38560	chr10:82845733-82859008:-
ENSMUSG00000000730	1.178822138	0.04358721	0.99977258	up	Dnmt3l	chr10:78041947-78063622:+
ENSMUSG00000006445	1.178121998	0.046115344	0.99977258	up	Epha2	chr4:141301240-141329384:+
ENSMUSG00000062488	1.17582604	0.002822499	0.532057228	up	Ifit3b	chr19:34607970-34613401:+
ENSMUSG00000017737	1.17489915	0.035156444	0.99977258	up	Mmp9	chr2:164940780-164955850:+
ENSMUSG00000090125	1.174137906	0.022595569	0.99977258	up	Pou3f1	chr4:124656807-124660655:+
ENSMUSG00000102329	1.171676733	0.024990732	0.99977258	up	Gm10851	chr2:11521706-11530836:+
ENSMUSG00000026102	1.170855432	0.003439504	0.563071516	up	Inpp1	chr1:52785427-52817688:-
ENSMUSG00000030516	1.162132947	0.00045875	0.227219987	up	Tjp1	chr7:65296165-65527781:-
ENSMUSG00000041754	1.160290478	0.032668632	0.99977258	up	Trem3	chr17:48247777-48258841:+
ENSMUSG00000025804	1.157529708	0.006188866	0.69893235	up	Ccr1	chr9:123962124-123968692:-
ENSMUSG00000003484	1.156577526	0.006638778	0.711607583	up	Cyp4f18	chr8:71988482-72009626:-
ENSMUSG00000024087	1.156452627	0.005228519	0.678728727	up	Cyp1b1	chr17:79706953-79715041:-
ENSMUSG00000030148	1.147027191	0.031651093	0.99977258	up	Clec4a2	chr6:123106428-123143999:+
ENSMUSG00000034674	1.143670309	0.003176022	0.549868097	up	Tdg	chr10:82629828-82650799:+
ENSMUSG00000030770	1.141629489	0.025902427	0.99977258	up	Parva	chr7:112427505-112591692:+
ENSMUSG00000098320	1.136395801	0.021490013	0.99977258	up	Vis1	chr2:45280724-45280924:+
ENSMUSG00000039994	1.135467672	0.023015614	0.99977258	up	Timeless	chr10:128232065-128252941:+
ENSMUSG00000032841	1.134541598	0.029090946	0.99977258	up	Prr5l	chr2:101714285-101883027:-
ENSMUSG00000026837	1.130298916	0.031233608	0.99977258	up	Col5a1	chr2:27886425-28039514:+
ENSMUSG00000052727	1.127682817	0.004293702	0.617836897	up	Map1b	chr13:99421446-99516540:-
ENSMUSG00000029380	1.127485095	0.029466732	0.99977258	up	Cxcl1	chr5:90891241-90893115:+
ENSMUSG00000055632	1.125896572	0.035122315	0.99977258	up	Hmcn2	chr2:31314415-31460738:+
ENSMUSG00000039377	1.12428481	0.011429056	0.882417961	up	Hlx	chr1:184727140-184732619:-
ENSMUSG00000017002	1.119530936	0.039968025	0.99977258	up	Slpi	chr2:164354070-164389095:-
ENSMUSG00000050945	1.114830573	0.034727465	0.99977258	up	Zfp438	chr18:5210031-5334439:-
ENSMUSG00000001663	1.114568992	0.042907087	0.99977258	up	Gstt1	chr10:75783813-75798584:-
ENSMUSG00000027381	1.111632728	0.020504063	0.99977258	up	Bcl2l11	chr2:128126038-128162547:+
ENSMUSG00000037946	1.109220487	0.014582121	0.948995202	up	Fgd3	chr13:49261554-49320311:-
ENSMUSG00000089999	1.106438194	0.022288534	0.99977258	up	Gm6485	chr3:104752727-104753165:-
ENSMUSG00000020473	1.104925981	0.040228138	0.99977258	up	Aebp1	chr11:5861947-5872088:+

ENSMUSG00000020641	1.103117057	0.029506772	0.99977258	up	Rsad2	chr12:26442746-26456452:-
ENSMUSG00000034394	1.099842334	0.029265582	0.99977258	up	Lif	chr11:4257557-4272514:+
ENSMUSG00000020032	1.092632196	0.03791575	0.99977258	up	Nuak1	chr10:84370905-84440597:-
ENSMUSG00000029699	1.091947948	0.030964457	0.99977258	up	Ssc4d	chr5:135960211-135974531:-
ENSMUSG00000039981	1.089927988	0.036851708	0.99977258	up	Zc3h12d	chr10:7832470-7870396:+
ENSMUSG00000062232	1.088981686	0.012442332	0.923322375	up	Rapgef2	chr3:79062516-79286517:-
ENSMUSG00000051682	1.086706837	0.010894137	0.875803143	up	Treml4	chr17:48264295-48275360:+
ENSMUSG00000020044	1.081299461	0.034625002	0.99977258	up	Timp3	chr10:86300372-86349506:+
ENSMUSG00000087141	1.069390748	0.034209266	0.99977258	up	Plcxd2	chr16:45959263-46010218:-
ENSMUSG00000057346	1.068295973	0.001911462	0.43538861	up	Apol9a	chr15:77403789-77411080:-
ENSMUSG00000035783	1.050216793	0.032433052	0.99977258	up	Acta2	chr19:34241090-34255336:-
ENSMUSG00000019944	1.050123495	0.021937095	0.99977258	up	Rhobtb1	chr10:69151434-69291791:+
ENSMUSG00000040522	1.048897189	0.042556775	0.99977258	up	Tlr8	chrX:167242696-167264329:-
ENSMUSG00000016559	1.048801966	0.016746119	0.993012722	up	H3f3b	chr11:116021912-116027962:-
ENSMUSG00000040552	1.045569916	0.0494422	0.99977258	up	C3ar1	chr6:122847138-122856161:-
ENSMUSG00000027360	1.044915242	0.011427298	0.882417961	up	Hdc	chr2:126593667-126619299:-
ENSMUSG00000044434	1.043279136	0.043453854	0.99977258	up	Gm9791	chr3:34005017-34005463:-
ENSMUSG00000034855	1.041947692	0.006222203	0.69893235	up	Cxcl10	chr5:92346638-92348889:-
ENSMUSG00000027204	1.036021531	0.003185212	0.549868097	up	Fbn1	chr2:125300594-125507993:-
ENSMUSG00000027358	1.034032004	0.007857595	0.767050903	up	Bmp2	chr2:133552159-133562885:+
ENSMUSG00000035385	1.033296168	0.004110761	0.617836897	up	Ccl2	chr11:82035571-82037453:+
ENSMUSG00000027333	1.028104239	0.012817482	0.928172059	up	Smox	chr2:131491496-131525922:+
ENSMUSG00000062345	1.02617623	0.03589658	0.99977258	up	Serpinb2	chr1:107511423-107535478:+
ENSMUSG00000054717	1.0181391	0.037164117	0.99977258	up	Hmgb2	chr8:57511907-57515999:+
ENSMUSG00000032311	1.017939536	0.046635979	0.99977258	up	Nrg4	chr9:55220222-55326844:-
ENSMUSG00000055723	1.0096605	0.037001286	0.99977258	up	Rras2	chr7:114046782-114117781:-
ENSMUSG00000025161	1.006591868	0.034780614	0.99977258	up	Slc16a3	chr11:120948480-120960868:+
ENSMUSG00000008393	1.004708184	0.025368584	0.99977258	up	Carhsp1	chr16:8658587-8672153:-
ENSMUSG00000021704	-1.008725259	0.013872056	0.940089742	down	Mtx3	chr13:92844760-92858230:+
ENSMUSG00000041921	-1.009276127	0.044893644	0.99977258	down	Metap1d	chr2:71453276-71525194:+
ENSMUSG00000087281	-1.010357458	0.014103367	0.941354027	down	Gm16015	chr5:43909215-43909885:-
ENSMUSG00000072568	-1.012851718	0.042751069	0.99977258	down	Fam84b	chr15:60818994-60853778:-
ENSMUSG00000021474	-1.017223194	0.017444699	0.994763824	down	Sfxn1	chr13:54071869-54108342:+
ENSMUSG00000085894	-1.032929265	0.007784582	0.764473907	down	Gm15832	chr1:39547570-39552002:-
ENSMUSG00000001053	-1.04005483	0.009632167	0.830928841	down	N4bp3	chr11:51643063-51650842:-
ENSMUSG00000026980	-1.046439679	0.016643308	0.993012722	down	Ly75	chr2:60292103-60383303:-
ENSMUSG00000022299	-1.053524509	0.035197537	0.99977258	down	Slc25a32	chr15:39091231-39112716:-
ENSMUSG00000100658	-1.056439804	0.024579811	0.99977258	down	F730311O21Rik	chr1:132342023-132349626:+
ENSMUSG00000024962	-1.058059469	0.017191829	0.99313989	down	Vegfb	chr19:6982473-6987651:-
ENSMUSG00000023009	-1.059097178	0.016775798	0.993012722	down	Nckap5l	chr15:99422035-99457748:-
ENSMUSG00000001918	-1.063292095	0.007154757	0.742645681	down	Slc1a5	chr7:16781340-16798274:+
ENSMUSG00000028238	-1.072488172	0.038689833	0.99977258	down	Atp6v0d2	chr4:19876841-19922605:-

ENSMUSG00000027082	-1.080872236	0.011339375	0.882417961	down	Tfpi	chr2:84432855-84476775:-
ENSMUSG00000058013	-1.094084312	0.013549786	0.928172059	down	10-Sep	chr5:93093437-93176447:+
ENSMUSG00000028811	-1.101189754	0.021411408	0.99977258	down	Yars	chr4:129189760-129219607:+
ENSMUSG00000017144	-1.102307935	0.033232451	0.99977258	down	Rnd3	chr2:51130438-51149111:-
ENSMUSG00000026389	-1.103949562	0.001053235	0.345461209	down	Steap3	chr1:120190757-120272705:-
ENSMUSG00000077704	-1.110992782	0.014481559	0.946205477	down	Snord89	chr1:39548745-39548854:-
ENSMUSG00000036362	-1.113403423	0.023639367	0.99977258	down	P2ry13	chr3:59207892-59210882:-
ENSMUSG00000023393	-1.114559156	0.02185309	0.99977258	down	Slc17a9	chr2:180725263-180742280:+
ENSMUSG00000027074	-1.114977393	0.0402473	0.99977258	down	Slc43a3	chr2:84936579-84958509:+
ENSMUSG00000115624	-1.11733865	0.037995114	0.99977258	down	AC154640.4	chr14:49023930-49028569:-
ENSMUSG00000060798	-1.117432004	0.009360131	0.823582002	down	Intu	chr3:40531286-40704774:+
ENSMUSG00000036661	-1.131849529	0.011675896	0.882417961	down	Dennd3	chr15:73512560-73572242:+
ENSMUSG00000003531	-1.135114496	0.04583745	0.99977258	down	Dgcr6	chr16:18052860-18071632:+
ENSMUSG00000098055	-1.137781285	0.008120652	0.788039625	down	Gm26947	chr10:60931991-60940942:+
ENSMUSG00000092274	-1.138201445	0.001456793	0.398190151	down	Neat1	chr19:5824708-5845478:-
ENSMUSG00000026637	-1.143209055	0.019986955	0.99977258	down	Traf5	chr1:191997205-192092559:-
ENSMUSG00000026873	-1.147736726	0.02534083	0.99977258	down	Phf19	chr2:34893757-34914026:-
ENSMUSG00000045875	-1.148272819	0.015879262	0.971716027	down	Adra1a	chr14:66635251-66771168:+
ENSMUSG00000006205	-1.148733257	0.025353903	0.99977258	down	Htra1	chr7:130936111-130985660:+
ENSMUSG00000102302	-1.165442591	0.042749204	0.99977258	down	Gm38190	chr1:165230371-165235125:-
ENSMUSG00000034487	-1.181175796	0.026884186	0.99977258	down	Kdelc2	chr9:53384025-53401867:+
ENSMUSG00000007029	-1.185507427	0.01893709	0.99977258	down	Vars	chr17:35000987-35016322:+
ENSMUSG00000096967	-1.187858497	0.023492756	0.99977258	down	Gm26621	chr15:68334715-68337430:-
ENSMUSG00000084911	-1.188528979	0.007721752	0.764473907	down	Gm16185	chr9:97085326-97088610:+
ENSMUSG00000025227	-1.19499779	0.018257007	0.99977258	down	Mfsd13a	chr19:46341121-46375252:+
ENSMUSG00000040618	-1.19962845	0.031685743	0.99977258	down	Pck2	chr14:55540266-55551242:+
ENSMUSG00000079038	-1.208036942	0.012644545	0.925761337	down	D130040H23Rik	chr8:69271080-69314207:+
ENSMUSG00000069516	-1.232185817	0.005125958	0.674593974	down	Lyz2	chr10:117277331-117282321:-
ENSMUSG00000092981	-1.23499514	0.008482583	0.804129224	down	Mir5125	chr17:23823291-23823369:+
ENSMUSG00000074758	-1.236594862	0.043861781	0.99977258	down	Gm5535	chr2:144098815-144207990:-
ENSMUSG00000022504	-1.237274877	0.022747498	0.99977258	down	Ciita	chr16:10488178-10527564:+
ENSMUSG00000047875	-1.239152607	0.00554571	0.678728727	down	Gpr157	chr4:150087365-150105927:+
ENSMUSG00000025094	-1.246780985	0.020388138	0.99977258	down	Slc18a2	chr19:59260878-59296012:+
ENSMUSG00000085396	-1.24789439	0.000448588	0.227219987	down	Firre	chrX:50555744-50635321:-
ENSMUSG00000035186	-1.262947604	0.015165119	0.967242849	down	Ubd	chr17:37193892-37196095:+
ENSMUSG00000026365	-1.279038148	0.013546814	0.928172059	down	Cfh	chr1:140084708-140183764:-
ENSMUSG00000087623	-1.281479702	0.005080326	0.674593974	down	Gm12404	chr4:41520694-41525823:+
ENSMUSG00000029491	-1.282263769	0.039953972	0.99977258	down	Pde6b	chr5:108388391-108432397:+
ENSMUSG00000031700	-1.290646533	0.011158306	0.880814703	down	Gpt2	chr8:85492576-85527560:+
ENSMUSG00000037999	-1.298907872	0.004304011	0.617836897	down	Arap2	chr5:62602445-62766159:-
ENSMUSG00000023809	-1.308602742	0.011465504	0.882417961	down	Rps6ka2	chr17:7170115-7303313:+
ENSMUSG00000109836	-1.315776906	0.010986986	0.878958895	down	Gm45884	chr18:36292626-36296041:+
ENSMUSG00000004267	-1.323892956	0.000471066	0.227219987	down	Eno2	chr6:124760053-124769673:-

ENSMUSG00000097134	-1.329204674	0.008562107	0.807003176	down	1110002J07Rik	chr10:66912489-66920258:-
ENSMUSG00000032013	-1.330768426	0.00315422	0.549868097	down	Trim29	chr9:43310848-43336115:+
ENSMUSG00000023045	-1.337313313	0.015536317	0.967242849	down	Soat2	chr15:102150526-102163474:+
ENSMUSG00000058581	-1.339850851	0.02561691	0.99977258	down	Gm5801	chr14:56010536-56011315:-
ENSMUSG00000040710	-1.342031068	0.004599476	0.632356235	down	St8sia4	chr1:95587682-95667571:-
ENSMUSG00000074093	-1.343644086	0.038986137	0.99977258	down	Svip	chr7:51997171-52006018:-
ENSMUSG00000042766	-1.352765642	0.025354352	0.99977258	down	Trim46	chr3:89234177-89246309:-
ENSMUSG00000085684	-1.3533905	0.041456359	0.99977258	down	4930469K13Rik	chr11:33973961-34098554:-
ENSMUSG00000030329	-1.363301337	0.000825951	0.306988867	down	Pianp	chr6:124996694-125003096:+
ENSMUSG00000028957	-1.367309116	0.000937588	0.327158407	down	Per3	chr4:151003652-151044665:-
ENSMUSG00000029919	-1.372373306	0.001165163	0.366715835	down	Hpgds	chr6:65117293-65144908:-
ENSMUSG00000039976	-1.374691776	0.014047775	0.941354027	down	Tbc1d16	chr11:119143045-119228499:-
ENSMUSG00000107756	-1.375678903	0.024731963	0.99977258	down	Gm44164	chr6:145238667-145241227:-
ENSMUSG00000115739	-1.378748962	0.036397805	0.99977258	down	AC101945.4	chr15:40012803-40014778:-
ENSMUSG00000037868	-1.387697119	0.003937277	0.617836897	down	Egr2	chr10:67535475-67542188:+
ENSMUSG00000060429	-1.390445722	0.016435736	0.990978177	down	Sntb1	chr15:55636388-55906949:-
ENSMUSG00000030117	-1.391304258	0.02363002	0.99977258	down	Gdf3	chr6:122605403-122610087:-
ENSMUSG00000057329	-1.399348835	0.002173934	0.454774515	down	Bcl2	chr1:106538178-106714274:-
ENSMUSG00000103509	-1.403625822	0.000429038	0.227219987	down	Gm38372	chr1:7148110-7152137:+
ENSMUSG00000042249	-1.408675801	0.000114941	0.099211855	down	Grk3	chr5:112910482-113015791:-
ENSMUSG00000052563	-1.411443373	0.024957998	0.99977258	down	D930048N14Rik	chr11:51650954-51657681:+
ENSMUSG00000107962	-1.454971278	0.010264503	0.854506837	down	Gm43980	chr6:49254851-49256462:+
ENSMUSG00000035064	-1.472460034	0.025301099	0.99977258	down	Eef2k	chr7:120842831-120907450:+
ENSMUSG00000026866	-1.474707784	0.006343132	0.702217616	down	Kynu	chr2:43555329-43682715:+
ENSMUSG00000027956	-1.488004694	0.029353482	0.99977258	down	Tmem144	chr3:79812564-79852773:-
ENSMUSG00000115276	-1.493257079	0.01412031	0.941354027	down	AC149588.1	chr15:34126944-34130688:+
ENSMUSG00000105632	-1.498376325	0.044131551	0.99977258	down	Gm43272	chr5:101810492-101812075:+
ENSMUSG00000105677	-1.500605474	0.001866214	0.4310691	down	Gm43328	chr3:105732444-105733877:-
ENSMUSG00000056014	-1.513586829	0.002030098	0.440552524	down	A430033K04Rik	chr5:138622859-138652414:+
ENSMUSG00000109539	-1.529652676	0.04693838	0.99977258	down	Gm44667	chr7:67268906-67272242:-
ENSMUSG00000081944	-1.534313254	0.011549556	0.882417961	down	Gm12276	chr11:62331421-62331721:+
ENSMUSG00000090957	-1.539778306	0.036102822	0.99977258	down	Gm7535	chr17:17911039-17911947:-
ENSMUSG00000108825	-1.542425925	0.002263242	0.463964598	down	Gm45838	chr7:83633371-83635817:+
ENSMUSG00000083257	-1.552644957	0.009568773	0.830306193	down	Gm15568	chr10:10343115-10344327:+
ENSMUSG00000107622	-1.55642877	1.45E-05	0.018298953	down	4930512J16Rik	chr6:89269511-89285725:-
ENSMUSG00000105572	-1.58336808	0.002426576	0.479468024	down	Gm43300	chr3:119727748-119729790:-
ENSMUSG00000066510	-1.58419413	0.000649411	0.266258556	down	Ankdd1a	chr9:65488470-65520193:-
ENSMUSG00000024620	-1.601984777	0.035500433	0.99977258	down	Pdgfrb	chr18:61045150-61085061:+
ENSMUSG00000107894	-1.609064585	0.049255609	0.99977258	down	Gm10388	chr6:149283838-149286773:-
ENSMUSG00000027901	-1.615986539	0.006467349	0.707096807	down	Dennd2d	chr3:106482405-106503030:+
ENSMUSG00000021365	-1.625379812	0.012017856	0.899967275	down	Nedd9	chr13:41309581-41487362:-
ENSMUSG00000039126	-1.6463761	0.02903196	0.99977258	down	Prune2	chr19:16956118-17223932:+

ENSMUSG00000114995	-1.664554463	0.009778614	0.830928841	down	AC137842.1	chr15:31621972-31624035:+
ENSMUSG00000105368	-1.667629452	0.018803101	0.99977258	down	Gm43759	chr5:112367212-112368472:+
ENSMUSG00000054000	-1.671582582	0.000416748	0.227219987	down	Tusc1	chr4:93334138-93335511:-
ENSMUSG00000022629	-1.674337267	0.041395579	0.99977258	down	Kif21a	chr15:90933276-91049948:-
ENSMUSG00000084834	-1.678385312	0.006635202	0.711607583	down	4930565N06Rik	chr16:36880519-36897189:-
ENSMUSG00000105556	-1.679283994	0.01430636	0.943352482	down	Gm43080	chr3:35969384-35972026:-
ENSMUSG00000027676	-1.684075814	0.010598261	0.86691639	down	Ccdc39	chr3:33812362-33844310:-
ENSMUSG00000100937	-1.716179586	0.045490978	0.99977258	down	1700020D05Rik	chr19:5502767-5503787:-
ENSMUSG00000030806	-1.717887118	0.040873309	0.99977258	down	Stx1b	chr7:127803900-127824549:-
ENSMUSG00000079484	-1.721162755	0.044773926	0.99977258	down	Phyhd1	chr2:30266203-30282149:+
ENSMUSG00000078700	-1.733017995	0.033502347	0.99977258	down	D030028A08Rik	chr11:96916270-96965060:+
ENSMUSG00000033949	-1.741246942	0.00405931	0.617836897	down	Trim36	chr18:46165302-46212607:-
ENSMUSG00000108053	-1.74902912	0.016249166	0.986986402	down	Gm43890	chr6:83314145-83316943:-
ENSMUSG00000025403	-1.750473275	0.006082777	0.693675995	down	Shmt2	chr10:127517123-127522444:-
ENSMUSG00000073427	-1.784038145	0.027885363	0.99977258	down	Gm4924	chr10:82354255-82422882:+
ENSMUSG00000105748	-1.785325823	0.029903052	0.99977258	down	Gm43088	chr3:137897144-137899490:+
ENSMUSG00000101609	-1.789478206	0.001412254	0.398190151	down	Kcnq1ot1	chr7:143212155-143296549:-
ENSMUSG00000097879	-1.807700591	0.006292539	0.702024758	down	Gm26869	chr2:178023808-178028736:-
ENSMUSG00000032827	-1.818673986	0.028226008	0.99977258	down	Ppp1r9a	chr6:4902917-5165661:+
ENSMUSG00000089988	-1.825884715	0.021704812	0.99977258	down	Gm16238	chr3:127691935-127692520:-
ENSMUSG00000029275	-1.83171245	0.045480269	0.99977258	down	Gfi1	chr5:107716657-107726036:-
ENSMUSG00000085913	-1.841814838	0.0016486	0.409062651	down	Gm15601	chr14:45563834-45598512:+
ENSMUSG00000115148	-1.853926191	0.005780044	0.688898751	down	AC107711.1	chr14:45612772-45614066:-
ENSMUSG00000072720	-1.863598746	0.009350901	0.823582002	down	Myo18b	chr5:112688876-112896362:-
ENSMUSG00000102559	-1.905043732	0.003076576	0.549868097	down	Gm37570	chr1:66765626-66768731:-
ENSMUSG00000032589	-1.916539522	0.019521054	0.99977258	down	Bsn	chr9:108096022-108190384:-
ENSMUSG00000103973	-1.927982335	0.01393799	0.940670897	down	BC055308	chr1:95328681-95331218:+
ENSMUSG00000108573	-1.944634511	0.037904445	0.99977258	down	Gm44986	chr7:122096083-122098911:+
ENSMUSG00000039057	-1.947661552	0.026563587	0.99977258	down	Myo16	chr8:10153911-10634742:+
ENSMUSG00000041313	-1.948506437	0.000846782	0.306988867	down	Slc7a1	chr5:148327410-148399904:-
ENSMUSG00000025007	-1.981682737	0.001536055	0.409062651	down	Aldh18a1	chr19:40550257-40588463:-
ENSMUSG00000078144	-2.0211674	0.024868142	0.99977258	down	Capns2	chr8:92901407-92902411:+
ENSMUSG00000103747	-2.052053769	0.048747481	0.99977258	down	Gm38236	chr2:68906003-68909231:+
ENSMUSG00000038740	-2.073273484	0.009130205	0.823582002	down	Mvb12b	chr2:33729953-33887946:-
ENSMUSG00000102662	-2.127679633	0.025702934	0.99977258	down	Gm38377	chr2:69018096-69021867:+
ENSMUSG00000016283	-2.135006569	0.005922661	0.688898751	down	H2-M2	chr17:37480851-37483552:-
ENSMUSG00000108954	-2.138094247	0.012606068	0.925761337	down	Gm44901	chr7:121052345-121056321:+
ENSMUSG00000070504	-2.138690608	0.023457557	0.99977258	down	Fcrl6	chr1:172596642-172602551:-
ENSMUSG00000039943	-2.162064397	0.044979355	0.99977258	down	Plcb4	chr2:135659011-136014593:+
ENSMUSG00000106757	-2.177986265	0.046649754	0.99977258	down	Gm43482	chr5:129964366-129966226:+
ENSMUSG00000040010	-2.178198355	0.034733612	0.99977258	down	Slc7a5	chr8:121881150-121907694:-
ENSMUSG00000097993	-2.184668681	2.76E-07	0.000645575	down	Ptprv	chr1:135108497-135132594:-
ENSMUSG00000091575	-2.224243181	0.001438315	0.398190151	down	2010016I18Rik	chr3:106481982-106485913:-

ENSMUSG00000107732	-2.236479396	0.038570818	0.99977258	down	Gm44204	chr6:89257820-89259136:-
ENSMUSG00000033762	-2.252329803	0.024674325	0.99977258	down	Recql4	chr15:76703553-76710559:-
ENSMUSG00000106108	-2.259088303	0.001185118	0.366715835	down	Gm43221	chr3:109000024-109002094:+
ENSMUSG00000029752	-2.300452414	0.042899166	0.99977258	down	Asns	chr6:7675169-7693254:-
ENSMUSG00000027896	-2.314871478	0.03126535	0.99977258	down	Slc16a4	chr3:107291230-107312115:+
ENSMUSG00000104291	-2.359976817	0.046235046	0.99977258	down	A130071D04Rik	chr1:138161820-138167683:-
ENSMUSG00000087799	-2.370329518	0.030058908	0.99977258	down	Gm25961	chr6:89249468-89249602:-
ENSMUSG00000104350	-2.390692747	0.011171308	0.880814703	down	Gm38244	chr3:106478283-106480868:+
ENSMUSG00000095419	-2.404645695	7.53E-05	0.08025484	down	Gm14328	chr18:74303524-74303931:-
ENSMUSG00000035829	-2.450565869	0.041020824	0.99977258	down	Ppp1r26	chr2:28446800-28455508:+
ENSMUSG00000063953	-2.482330673	0.000279936	0.176574875	down	Amd2	chr10:35708675-35711892:-
ENSMUSG00000024640	-2.502070843	0.020185696	0.99977258	down	Psat1	chr19:15904678-15947337:-
ENSMUSG00000105304	-2.532768174	0.04210105	0.99977258	down	Gm43696	chr3:104470660-104476730:-
ENSMUSG00000103845	-2.540046127	0.002055283	0.440552524	down	Gm19026	chr1:6429655-6441296:-
ENSMUSG00000025932	-2.608813324	0.023595015	0.99977258	down	Eya1	chr1:14168954-14310235:-
ENSMUSG00000027313	-2.661427294	0.030809046	0.99977258	down	Chac1	chr2:119351229-119354381:+
ENSMUSG00000112067	-2.675177934	0.038878078	0.99977258	down	AC125487.1	chr12:95734110-95735409:+
ENSMUSG00000102305	-2.720178039	0.022078114	0.99977258	down	Gm38192	chr9:73024293-73026139:+
ENSMUSG00000085666	-2.775566703	2.63E-08	8.63E-05	down	Gm9855	chr10:42054113-42055306:-
ENSMUSG00000026023	-2.795938818	0.023931954	0.99977258	down	Cdk15	chr1:59256906-59352993:+
ENSMUSG00000004552	-2.824907268	1.34E-06	0.002204959	down	Ctse	chr1:131638306-131675505:+
ENSMUSG00000109679	-2.879382674	0.01752968	0.994763824	down	Gm45342	chr8:25692146-25693508:+
ENSMUSG00000113326	-2.881434236	0.047324471	0.99977258	down	CT009757.2	chr13:63244221-63246248:+
ENSMUSG00000099413	-2.890705222	0.022637987	0.99977258	down	Gm17767	chr1:51507086-51596718:-
ENSMUSG00000097914	-2.96668169	0.031149838	0.99977258	down	Gm26838	chr16:35805947-35808946:-
ENSMUSG00000062545	-2.97349742	0.017198276	0.99313989	down	Tlr12	chr4:128615443-128618619:-
ENSMUSG00000113041	-2.976927832	0.033562245	0.99977258	down	CT025556.1	chr13:75727279-75729162:+
ENSMUSG00000109784	-2.983205299	0.010208719	0.854198954	down	Gm45493	chr8:18862196-18866143:+
ENSMUSG00000103291	-3.046019035	0.01009247	0.848802638	down	Gm38235	chr1:106219314-106224265:+
ENSMUSG00000085663	-3.260281993	0.034050457	0.99977258	down	Gm15718	chr7:132979582-132983552:-
ENSMUSG00000033849	-3.278550058	0.015475252	0.967242849	down	B3galt2	chr1:143640664-143654614:+
ENSMUSG00000070547	-3.321591558	0.048911522	0.99977258	down	Mrgprb1	chr7:48444113-48456342:-
ENSMUSG00000084252	-3.472396743	0.024846755	0.99977258	down	Gm12036	chr11:20729420-20729949:+
ENSMUSG00000074280	-3.502892418	0.000636525	0.266258556	down	Gm6166	chr9:57483963-57484371:+
ENSMUSG00000033032	-3.507445464	0.026893024	0.99977258	down	Afap1l1	chr18:61730261-61786702:-
ENSMUSG00000104262	-3.658976836	0.019096119	0.99977258	down	Gm37747	chr2:68989381-68993383:+
ENSMUSG00000112141	-3.672736124	0.047188729	0.99977258	down	4930563J15Rik	chr10:67568522-67571989:-
ENSMUSG00000079445	-3.684254513	0.00016588	0.129544504	down	B3gnt7	chr1:86302832-86307305:+
ENSMUSG00000084930	-3.739048719	0.037377314	0.99977258	down	Gm11706	chr11:106814670-106816008:-
ENSMUSG00000053398	-3.760429351	0.02751212	0.99977258	down	Phgdh	chr3:98313170-98339990:-
ENSMUSG00000104509	-3.9444124	0.034050714	0.99977258	down	Gm33994	chr1:136576938-136579477:-
ENSMUSG00000110569	-3.944508673	0.006946456	0.726895275	down	Gm18860	chr8:71704205-71704968:-
ENSMUSG00000101587	-4.002293016	0.030805934	0.99977258	down	Gm29036	chr5:45768867-45769743:-

ENSMUSG00000049612	-4.002715295	0.031228104	0.99977258	down	Omg	chr11:79500982-79504084:-
ENSMUSG00000115522	-4.1570224	0.019431429	0.99977258	down	AC107757.1	chr15:95802033-95804401:-
ENSMUSG00000108780	-4.272739352	0.026348565	0.99977258	down	5430434F05Rik	chr6:83417440-83420992:-
ENSMUSG00000051331	-4.274797193	0.048051785	0.99977258	down	Cacna1c	chr6:118587240-119196418:-
ENSMUSG00000081223	-4.281446153	0.004193068	0.617836897	down	Gm12247	chr11:58097104-58097773:-
ENSMUSG00000112981	-4.328914679	0.041418895	0.99977258	down	5033424D13Rik	chr12:108878769-108879416:+
ENSMUSG00000022085	-4.32900343	0.034374896	0.99977258	down	Pebp4	chr14:69840420-70059918:+
ENSMUSG00000080180	-4.32900343	0.034374896	0.99977258	down	Gm17235	chr11:78576237-78577355:+
ENSMUSG00000095178	-4.357909252	0.033231403	0.99977258	down	Gm22716	chr5:110677124-110677230:-
ENSMUSG00000111362	-4.358151499	0.040707115	0.99977258	down	AC153955.3	chr10:44715558-44717165:+
ENSMUSG00000033491	-4.416658139	0.044590839	0.99977258	down	Prss35	chr9:86743649-86758443:+
ENSMUSG00000028373	-4.436399891	0.020438157	0.99977258	down	Astn2	chr4:65380803-66404611:-
ENSMUSG00000104200	-4.571886667	0.043461284	0.99977258	down	Gm37399	chr3:51302488-51302903:-
ENSMUSG00000102451	-4.572315396	0.045920692	0.99977258	down	Gm37655	chr1:139082862-139086984:-
ENSMUSG00000102427	-4.572706905	0.048784912	0.99977258	down	Gm37463	chr1:177229891-177230686:-
ENSMUSG00000085787	-4.582981045	4.19E-08	0.00011447	down	Gm13092	chr4:150315177-150315436:-
ENSMUSG00000079286	-4.655778637	0.016522195	0.992542098	down	Gm11084	chr2:72971548-72986716:-
ENSMUSG00000027296	-4.656006424	0.01511297	0.967242849	down	Itpka	chr2:119742337-119751263:+
ENSMUSG00000000247	-4.718034885	0.003246362	0.550235128	down	Lhx2	chr2:38339281-38369733:+
ENSMUSG00000094509	-4.773781775	0.038758892	0.99977258	down	Ighv14-1	chr12:113931953-113932382:-
ENSMUSG00000009545	-4.8447764	0.023607626	0.99977258	down	Kcnq1	chr7:143106362-143427042:+
ENSMUSG00000104235	-4.945347097	0.008226322	0.793598118	down	Gm37589	chr3:60535751-60539744:+
ENSMUSG00000105457	-4.987708579	0.01794715	0.99977258	down	Gm43200	chr5:45770020-45772180:-
ENSMUSG00000103546	-4.987977015	0.018722044	0.99977258	down	Gm37666	chr2:69013612-69014477:+
ENSMUSG00000101431	-5.025457343	0.004605975	0.632356235	down	Gm7901	chr8:75711600-75713196:-
ENSMUSG00000103706	-5.32953592	0.00350203	0.563071516	down	6820402A03Rik	chr1:66793645-66796298:-
ENSMUSG00000042684	-5.445759694	0.000234143	0.166954246	down	Npl	chr1:153503015-153550045:-
ENSMUSG00000015890	-5.817750319	7.83E-05	0.08025484	down	Amdhd1	chr10:93523338-93540033:-
ENSMUSG00000064246	-7.365552139	3.02E-62	4.96E-58	down	Chil1	chr1:134182176-134190181:+
ENSMUSG00000020017	-8.407370486	8.16E-56	6.69E-52	down	Hal	chr10:93488768-93519304:+
ENSMUSG00000062082	-11.58436952	0.000630043	0.266258556	down	Cd200r4	chr16:44811733-44839150:+

Table S4. Primer sequences for RT-PCR

	Forward primer (5′-3′)	Reverse primer (5′-3′)
Gpr68	CGTGGTCATCTTCCTGGCTT	TGGTGAGGAGGAGGGAGAAG
Mmp9	GCCCTGGAACTCACACGACA	TTGGAAACTCACACGCCAGAAG
IL-1β	TCCAGGATGAGGACATGAGCAC	GAACGTCACACACCAGCAGGTTA
IL-6	CTGCAAGAGACTTCCATCCAG	AGTGGTATAGACAGGTCTGTTGG
Tnf-α	CTGAACTTGGGGGTGATCGG	GGCTTGTCACTCGAATTTTGAG
Tsp1	TGGCCAGCGTTGCCA	TCTGCAGCACCCCCTGAA
Mus β-actin	CATCCGTAAAGACCTCTATGCCAAC	ATGGAGCCACCGATCCACA
