# Supplementary figures and images for: Loss of myeloid Tsc2 predisposes to angiotensin II-induced aortic aneurysm formation in mice
Source: Cell Death Dis. 2022 Nov 18;13(11):972. doi: 10.1038/s41419-022-05423-2 (PMC9674579; doi:10.1038/s41419-022-05423-2)

**Fig 2D**

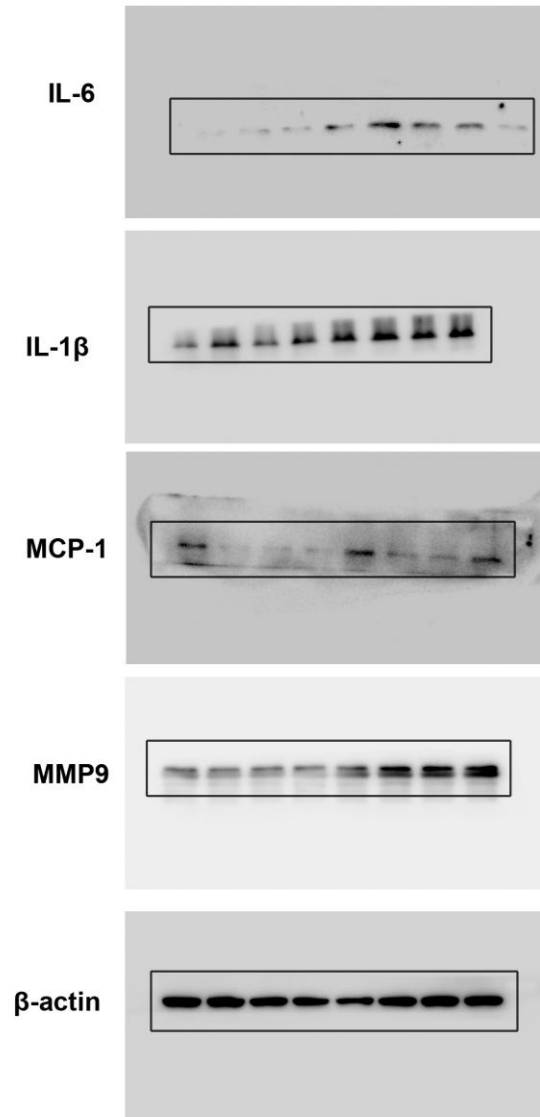

**Fig 4B**

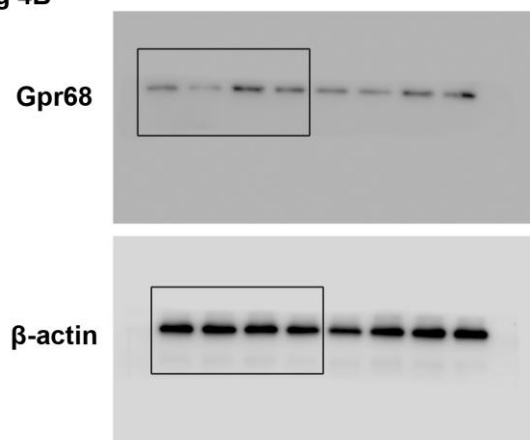

**Fig 5B**

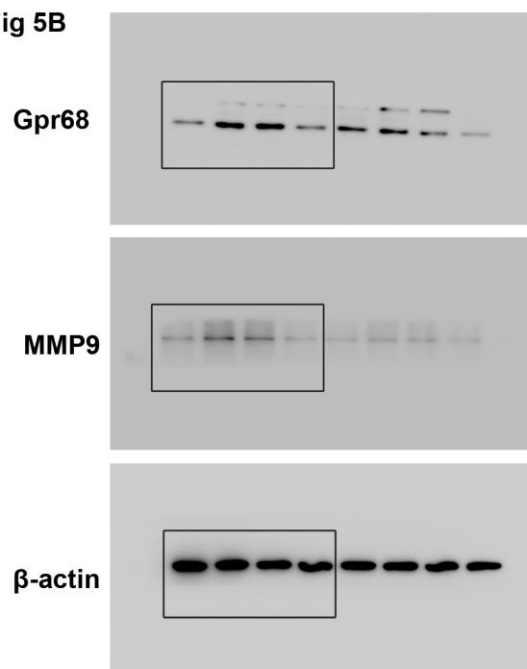

**Fig 5E**

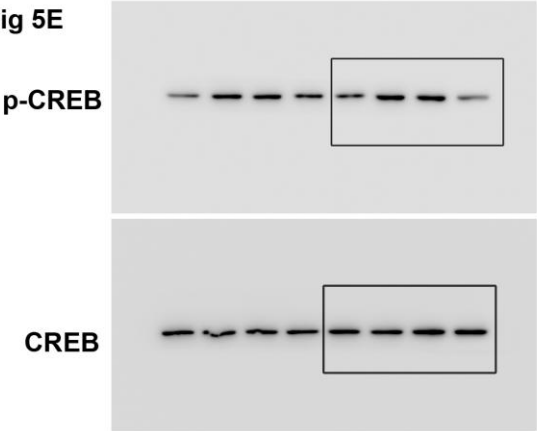

**FigS1**

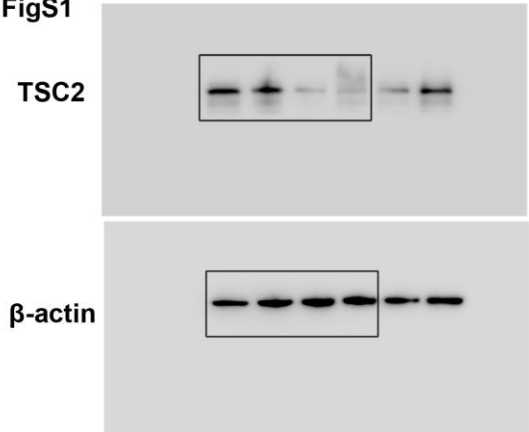

Supplement: Supplementary file 2 — Full and uncropped western blots [file 41419_2022_5423_MOESM2_ESM.pdf]
